# Single-cell RNA-sequencing identifies the developmental trajectory of C-Myc-dependent NK1.1^−^ T-bet^+^ intraepithelial lymphocyte precursors

**DOI:** 10.1038/s41385-019-0220-y

**Published:** 2019-11-11

**Authors:** Jonas F. Hummel, Patrice Zeis, Karolina Ebert, Jonas Fixemer, Philip Konrad, Christian Schachtrup, Sebastian J. Arnold, Dominic Grün, Yakup Tanriver

**Affiliations:** 1 Institute of Medical Microbiology and Hygiene, Faculty of Medicine, University of Freiburg, 79104 Freiburg, Germany; 2 Max Planck Institute of Immunobiology and Epigenetics, 79108 Freiburg, Germany; 3 Faculty of Biology, University of Freiburg, Schaenzlestrasse 1, 79104 Freiburg, Germany; 4 International Max Planck Research School for Molecular and Cellular Biology (IMPRS-MCB), Freiburg, Germany; 5 Institute of Anatomy and Cell Biology, Faculty of Medicine, University of Freiburg, 79104 Freiburg, Germany; 6 Institute of Experimental and Clinical Pharmacology and Toxicology, Faculty of Medicine, University of Freiburg, 79104 Freiburg, Germany; 7 CIBSS - Centre for Integrative Biological Signaling Studies, University of Freiburg, Freiburg, Germany; 8 Department of Internal Medicine IV, Medical Center – University of Freiburg, Faculty of Medicine, University of Freiburg, 79106 Freiburg, Germany

## Abstract

Natural intraepithelial lymphocytes (IELs) are thymus-derived adaptive immune cells, which are important contributors to intestinal immune homeostasis. Similar to other innate-like T cells, they are induced in the thymus through high-avidity interaction that would otherwise lead to clonal deletion in conventional CD4 and CD8 T cells. By applying single-cell RNA-sequencing (scRNA-seq) on a heterogeneous population of thymic CD4^−^CD8αβ^−^TCRαβ^+^NK1.1^−^ IEL precursors (NK1.1^−^ IELPs), we define a developmental trajectory that can be tracked based on the sequential expression of CD122 and T-bet. Moreover, we identify the Id proteins Id2 and Id3 as a novel regulator of IELP development and show that all NK1.1^−^ IELPs progress through a PD-1 stage that precedes the induction of T-bet. The transition from PD-1 to T-bet is regulated by the transcription factor C-Myc, which has far reaching effects on cell cycle, energy metabolism, and the translational machinery during IELP development. In summary, our results provide a high-resolution molecular framework for thymic IEL development of NK1.1^−^ IELPs and deepen our understanding of this still elusive cell type.

## Introduction

Intraepithelial lymphocytes (IELs) are an important component of the epithelial barrier that constitutes the boundary between the body and the environment. The intestine consists of various innate and adaptive immune cells that execute specific functions to maintain epithelial integrity and intestinal immune homeostasis.^1^ Here adaptive immune cells can be broadly divided into induced and natural IELs.^2^ Natural IELs comprise both T cell receptor (TCR) γδ^+^ and TCRαβ^+^ T cells, which lack the classical co-receptor CD4 or CD8αβ (double negative (DN)) but instead largely express the homodimer CD8αα. Natural TCRαβ^+^ IELs are selected and fate-determined in the thymus through high affinity TCR interaction with self-peptide major compatibility complex (MHC) in a process termed “agonist selection.”^3,4^ This pathway is not unique to natural TCRαβ^+^ IELs as other lineages, e.g., invariant natural killer T (NKT) cells and thymic regulatory T cells, also require strong TCR interactions for their development.^5,6^ In contrast, such strong interaction would result in the clonal deletion of conventional CD4 and CD8αβ single-positive (SP) T cells, which are selected by low affinity TCR stimulation.^7^

Strong agonist interaction in thymocytes correlates with the induction of several transcription factors (TFs; e.g., Helios, Nur77, and Egr2) and expression levels of surface molecules (e.g., programmed cell death protein 1 (PD-1), CD5, CD4, CD8αβ, and CD69).^8,9^ Of particular interest in this context is the induction of PD-1, which has been proposed as a unifying and discriminatory marker of thymocytes with a history of strong agonist selection. For example, αβTCRs cloned from intestinal natural IELs and re-expressed in a timely fashion during thymocyte development primarily gave rise to natural IELs.^10^ Moreover, the same study could show that, during thymic development, these cells sequentially lost CD4 and CD8αβ after positive selection and gained the expression of CD69, Nur77, Helios, Egr2, and PD-1.^10^ In support of these findings, another group identified thymic IEL precursors (IELPs) as CD4^−^CD8αβ^−^TCRαβ^+^Thy1^+^CD5^+^CD122^+^PD-1^+^.^11^ Finally, the expression of PD-1 marks autoreactive CD4^+^ T cells that are deleted via Bim-dependent apoptosis.^12^ In contrast, a more recent report used temporary fate mapping and SPADE (spanning-tree progression analysis of density-normalized events) analysis of flow cytometric data to propose that natural TCRαβ^+^ IELs are the progeny of two non-related thymic precursors.^13^ Intriguingly, one precursor population (named “type A” IELPs) was NK1.1^−^PD-1^+^T-bet^−^, whereas the other showed an opposite profile (named “type B” IELPs: NK1.1^+^PD-1^−^T-bet^+^). This new distinction was possible as the authors used CD1d tetramers to more precisely exclude NKT cells instead of the commonly used anti-NK1.1 antibody.^13^

In addition to fate determination, strong agonist selection in conjunction with interleukin (IL)-15 signaling induces the T-box TF T-bet, which plays a non-redundant role in proliferation and differentiation of IELPs.^14,15^ Similarly, TCR affinity and cytokine signaling are also important for activation of conventional T cells. These separate events are then integrated by the TF C-Myc,^16,17^ which connects T cell stimulation to cell cycle progression and proliferation, in parts through adaption of the cellular metabolism.^18^ Vice versa, T cell-specific knockouts of C-Myc are severely deficient for natural TCRαβ^+^ IELs.^19^ This phenotype is reminiscent of *Il15*^−^^*/*^^−^ and *Tbx21*^−^^*/*^^−^ mice;^14,20^ however, the role of C-Myc within this network in IELPs remained to be determined.

A fundamental problem of deciphering the pathway of IELP development is the cellular heterogeneity of thymic DN TCRαβ^+^ cells. So far, this issue has been mostly addressed by using TCR transgenic mouse systems and multi-color flow cytometry, which have given us valuable insights. However, both of these approaches have their intrinsic limitations, as they are reductionist approaches with a restricted TCR repertoire and a focus on “anticipated” targets, respectively. To overcome these obstacles, we combined a hypothesis-driven approach with the powerful tool of single-cell RNA-sequencing (scRNA-seq) and machine learning to gain unprecedented access to the stepwise process of thymic IEL development.^21^ scRNA-seq unraveled the developmental trajectory of thymic NK1.1^−^ IELPs, which can be tracked based on the sequential expression of CD122 and T-bet. Furthermore, we identify Id2 and Id3 as novel regulators of IELP development and show that all NK1.1^−^ IELPs progress through a PD-1 stage that precedes the induction of T-bet. We could further unravel that this transition is guided by the TF C-Myc, which not only regulates cell cycle but also has a profound impact on energy metabolism and overall protein synthesis. In summary, we provide a detailed picture of IEL development of NK1.1^−^ IELPs, which broadens and deepens our understanding of agonist-selected T cells.

## Results

### Single-cell transcriptomics reveal thymic differentiation pathway of NK1.1^−^ IELPs

**Fig. 1:**
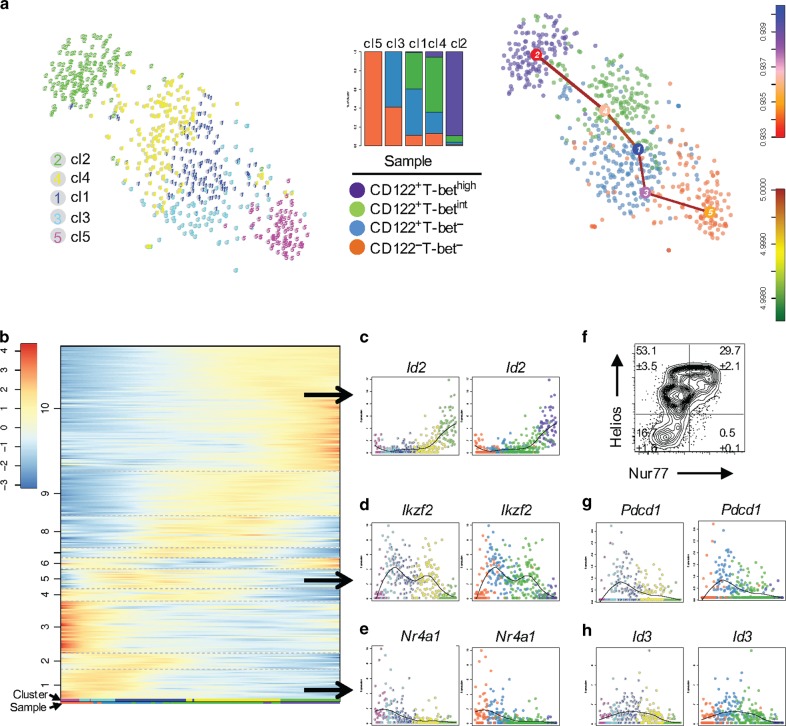
Single-cell transcriptomics reveal thymic differentiation pathway of IELPs. **a** *t*-distributed stochastic neighbor embedding (t-SNE) map representation of transcriptome similarities between 645 cells of 4-week-old TBGR mouse clustered by RaceID3 (left). Bar diagram showing the cumulative percentage of the sample contribution, normalized by sample size, to each cluster (mid). t-SNE map showing the sample information for RaceID3 clustered cells with an overlaid putative differentiation trajectory derived by StemID2 (right). **b** Self-organizing map (SOM) of *z*-score transformed pseudo-temporal expression profiles, grouped into 10 modules of co-expressed genes, along the putative differentiation trajectory (5→3→1→4→2). The pseudo-temporal order was inferred by StemID2-derived projection coordinates of all cells. The color codes below the SOM indicate the cluster information (upper panel) or sample information (lower panel). **c**–**e** Example expression profiles of the SOM-identified modules representing confirmed agonist-selection markers *Id2* (encodes for Id2) (**c**), *Ikzf2* (encodes for Helios) (**d**), and *Nr4a1* (encodes for Nur77) (**e**) along the predicted IELP differentiation trajectory from **b** with cluster information (left panel) and sample information (right panel). The black line in each plot indicates a local regression. **f** Correlation of agonist-selection markers Helios and Nur77 was also observed on protein level as shown by flow cytometric analysis of NK1.1^−^ IELPs from 6- to 12-week-old TBGR mice. Numbers denote the percentage of cells in the gate (Mean ± SEM) from three mice, experiments were performed twice, and one representative result is shown. **g**, **h** Expression profiles of *Pdcd1* (encodes for PD-1) (**g**) and *Id3* (encodes for Id3) (**h**) along the predicted IELP differentiation trajectory  from b with cluster information (left panel) and sample information (right panel). The black line in each plot indicates local regression

Historically, IELPs have been defined as DN TCRαβ^+^NK1.1^−^ thymocytes.^22^ However, recent reports have shown that based on TFs and surface molecules this population is highly diverse without an unambiguous differentiation model of natural TCRαβ^+^ IELs. This diversity might be explained by an undiscovered heterogeneity and plasticity, which can be revealed based on differences in gene expression. To characterize the heterogeneity and to uncover a developmental trajectory, we applied scRNA-seq on thymic DN TCRαβ^+^NK1.1^−^ IELPs (Supplementary Fig. 1a). We used CD4 and NK1.1. to exclude NKT cells from our sorting strategy (Supplementary Fig. 1a, b), hence the presented data primarily focuses on recently termed “type A” IELPs.^13^ Since not all NKT cells express NK1.1 in the thymus, we additionally stained bulk thymocytes with αGalCer:CD1d^+^ tetramer, which demonstrated that less than 3% of DN TCRαβ^+^NK1.1^−^ IELPs are potentially NKT precursors (Supplementary Fig. 1c). As shown before by us,^14,15^ NK1.1^−^ IELPs harbor a significant population of cells that express T-bet (Supplementary Fig. 1a). Since T-bet is a lineage-defining TF for IELs and only a minority of all IELPs express T-bet, we used 4-week-old T-bet ZsGreen reporter (TBGR) mice^23^ for index sorting of IELPs based on T-bet (i.e., ZsGreen) expression.^15^ We randomly sampled and sorted the following populations: CD122^−^T-bet^−^, CD122^+^T-bet^−^, CD122^+^T-bet^intermediate(int)^, and CD122^+^T-bet^high^ IELPs in equal numbers (Supplementary Fig. 1a), thus we enriched for T-bet^int^ and T-bet^high^ IELPs, as they would be underrepresented using an unbiased sorting strategy. With this strategy, we hypothesized that all developmental steps were captured, from which we could derive potential differentiation trajectories. After library preparation and sequencing, we obtained 645 high-quality RNA profiles of IELPs with at least 2000 transcripts per cell. Irrespective of the four sorted populations, total cells were then clustered using the RaceID3 algorithm^24^ and a putative lineage tree was derived using StemID2.^25^ We obtained five clusters, which form a single differentiation trajectory with no branching points (Fig. 1a). Next, we mapped the phenotype of our index sorted cells individually onto those clusters to test whether the development of IELPs can be tracked based on the expression of CD122 and T-bet. Clusters 5 and 2 represent the opposite ends of the inferred trajectory and almost solely consist of CD122^−^Tbet^−^ and CD122^+^T-bet^high^ cells, respectively (Fig. 1a, middle). Cluster 5 (CD122^−^T-bet^−^) establishes the root of the tree, consisting of the most undifferentiated cells, and cluster 2 (CD122^+^T-bet^high^) constitutes the end point (Fig. 1a), comprising mature IELPs expressing high levels of *Id2*,^26^ known to be upregulated by intestinal IELs (Fig. 1b, c), and NK cell markers^27^ such as *Klrb1c* and *Klrk1* (Supplementary Fig. 1e). For the other three clusters, only two significant inter-cluster links were inferred connecting these clusters to their previous and subsequent clusters on the predicted trajectory. Hence, on this trajectory cluster 3 succeeds cluster 5 and mainly consists of CD122^+^T-bet^−^ cells, followed by cluster 1, which comprises both CD122^+^T-bet^−^ and CD122^+^T-bet^int^ cells. The next stage of the predicted trajectory is cluster 4 with a decreased frequency of CD122^+^T-bet^−^ cells and a majority of CD122^+^T-bet^int^ cells, ultimately giving rise to cluster 2 (Fig. 1a, middle), i.e., cluster 5→3→1→4→2. Since the sorted populations do not cluster separately but intermingle within individual clusters, a technical batch effect arising from the sorting strategy is unlikely. Thus the predicted trajectory supports our previous hypothesis that putative CD122^−^T-bet^−^ early NK1.1.^−^ IELPs from the DN stage differentiate and go through a CD122^+^T-bet^−^ stage, followed by a CD122^+^T-bet^int^ stage to eventually become CD122^+^T-bet^high^ NK1.1^−^ IELPs (Fig. 1a, right).

As StemID2 allows the derivation of a pseudo-temporal order of cells from their positions on the inter-cluster links,^24,25^ we inferred pseudo-temporal expression profiles for all genes and investigated expression dynamics along the predicted trajectory. Utilizing self-organizing maps (SOM), we grouped these profiles into 10 modules of co-expressed genes along the predicted differentiation trajectory (Fig. 1b). Dynamic expression profile of *Tbx21* confirmed the faithfulness of the ZsGreen reporter in our study (Supplementary Fig. 1d). Previously, we demonstrated that prior to T-bet upregulation, IELPs are agonist-selected and express Helios.^14^ Thus we investigated the expression of agonist-selection markers along the predicted IELP differentiation trajectory. In agreement with our assumption, IELPs express *Ikzf2* (encodes for Helios) prior to *Tbx21* expression, starting in the CD122^−^T-bet^−^ stage and reaching its maximum in the CD122^+^T-bet^−^ stage (Fig. 1b, d). The decline in *Ikzf2* expression is accompanied by the onset of Id2 expression in cluster 4, mainly comprising CD122^+^T-bet^int^ cells, thus marking the commitment to the IEL lineage (Fig. 1c). In module 1, which is established slightly earlier than *Ikzf2* expression (module 5), we found other genes important for agonist selection of IELP such as *Nr4a1* (encodes for Nur77, Fig. 1b, e).^5^ This correlation was also evident on the protein level as shown by flow cytometry (Fig. 1f). With confidence in our predicted trajectory, we started to screen for genes with a pivotal role during IEL differentiation. Within the CD122^+^T-bet^−^ stage, we observed upregulation of *Pdcd1* (encodes for PD-1), which declines during the CD122^+^T-bet^int^ stage (Fig. 1g). Hence, NK1.1^−^ IELPs progress through a PD-1^+^ stage prior to the upregulation of T-bet. Interestingly, in the same module containing *Ikzf2*, we found the expression of *Id3*. *Id3* expression initiates at the CD122^−^T-bet^−^ stage and peaks in cluster 1, at the interface of the CD122^+^T-bet^−^ and the CD122^+^T-bet^int^ stage (Fig. 1h). Thus we anticipated that Id3 might play a crucial role during IELP development.

### Id3 regulates the pool size of thymic IELPs

Next, and to validate the power of our scRNA-seq derived developmental trajectory, we focused on the Id proteins Id2 and Id3. Id3 is a helix–loop–helix (HLH) TF that belongs to the family of Id proteins, which consists of four members in vertebrates (Id1, Id2, Id3, and Id4). Id proteins inhibit the transcriptional activity of another class of HLH proteins, the E-proteins, by forming heterodimeric complexes with the latter and thus inhibiting their DNA-binding capacity.^28^ In the immune system, E-proteins are crucial for lineage commitment, differentiation, and lymphocyte function. For example, in T cells Id3 is induced upon TCRβ selection at DN3b, remains high in naive T cells, and shows a bimodal pattern in memory T cells.^29,30^ As a result, Id3-deficient mice show defects in thymic development, steady-state function, and long-term memory formation of T cells.^29,30^ To interrogate the role of Id3 in IELs, we took advantage of Id3-GFP reporter mice, in which *Gfp* is inserted into the *Id3* locus and thus creating a functional null allele (*Id3*^*Gfp/+*^).^29^

**Fig. 2:**
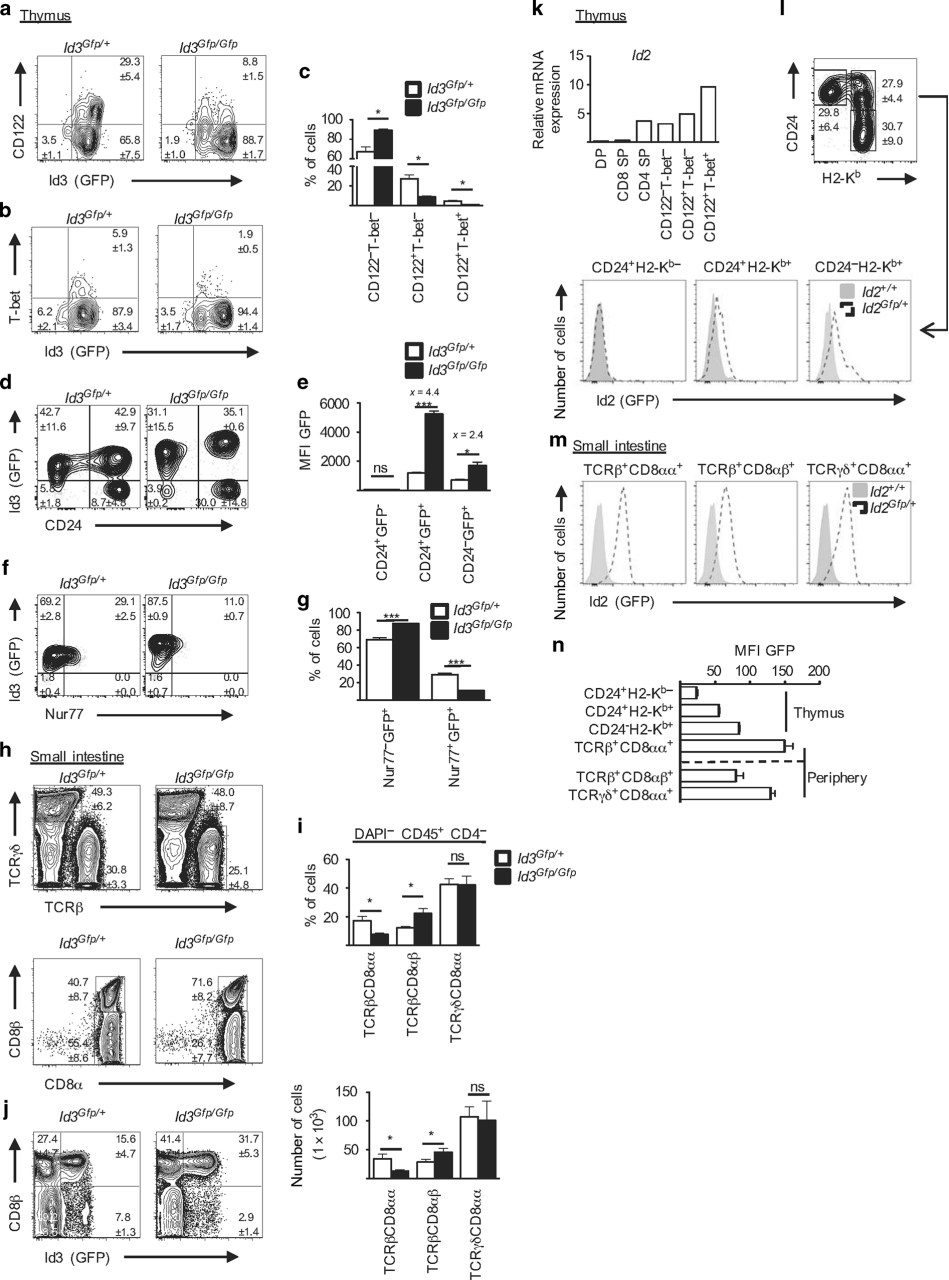
Id3 regulates the pool size of NK1.1^−^ IELPs. **a**, **b** Flow cytometric analysis of thymocytes of 6–12-week-old *Id3*^*Gfp/+*^ (littermate controls) and *Id3*^*Gfp/Gfp*^ mice. Cells were pre-gated on NK1.1^−^ IELPs as shown in Supplementary Fig. 1a and analyzed for Id3 (GFP) expression vs. CD122 (**a**) and T-bet (**b**). Bar diagram represents percentage (Mean ± SEM) of NK1.1^−^ IELP subpopulations based on CD122 and T-bet expression as in **a**, **b**. **d** Contour plot shows GFP vs. CD24 expression in thymic NK1.1^−^ IELP. **e** Bar diagram shows GFP MFI from **d**. **f** Contour plot shows GFP vs. Nur77 expression in thymic NK1.1^−^ IELPs. **g** Bar diagram shows percentage (Mean ± SEM) of the indicated populations as gated in **f**. **h** Upper row shows flow cytometric analysis of lymphocytes isolated from the epithelium of the small intestine of 6–12-week-old *Id3*^*Gfp/Gfp*^ mice with *Id3*^*Gfp/+*^ littermates as controls. Cells were pre-gated on DAPI^−^ CD45^+^ CD4^−^. Lower row shows further flow cytometric analysis of TCRβ^+^ IELs for CD8β^+^ and CD8α^+^. Numbers denote the percentage of cells in the gate (Mean ± SEM). **i** Bar diagrams represent percentages (upper, Mean ± SEM) and absolute cell numbers (lower, Mean ± SEM) of TCRβ^+^ and TCRγδ^+^ IELs as shown in **h**. **j** Dot plot shows GFP expression of small intestine TCRβ^+^ IELs gated as in **h**. **a**–**j** Experiments were performed twice with at least six mice per genotype. **k** Bar diagram represents mRNA expression of *Id2* for different thymocyte populations from three 6–12-week-old TBGR mice performed by qPCR. **l** Flow cytometric analysis of thymocytes of 4–8-week-old *Id2*^*Gfp/+*^ mice. Dot plot shows the expression of CD24 vs. H2-K^b^ maturation of thymic NK1.1^−^ IELPs and numbers denote the percentage of cells in the gate (Mean ± SEM) from four mice. Histogram represents MFI expression of GFP (Id2) of the indicated thymic NK1.1^−^ IELP populations for *Id2*^*Gfp/+*^ and *Id2*^*+/+*^ mice as controls. **m** Histogram represent MFI expression of GFP (Id2) of the indicated IEL populations in the small intestine for *Id2*^*Gfp/+*^ and *Id2*^*+/+*^ mice from one representative mouse. **n** Bar diagram shows MFI (Mean ± SEM) of GFP (Id2) in thymic NK1.1^−^ IELPs and small intestine IELs as shown in **l**, **m** from four mice

Similar to CD4 and CD8 SP T cells, most IELPs were GFP^+^ (i.e., Id3^+^) (Supplementary Fig. 2a). Careful analysis of IELPs from *Id3*^*Gfp/+*^ mice further revealed that there was a slight, but significant, increase of green fluorescent protein (GFP) expression when IELPs co-expressed CD122 (Fig. 2a). T-bet^+^ IELPs remained GFP^+^ (Fig. 2b). In contrast, IELPs in *Id3*^*Gfp/Gfp*^ mice (i.e., *Id3* knockout mice) expressed less CD122 (Fig. 2a) and as a result had a significant reduction in CD122^+^T-bet^+^ IELPs (Fig. 2c). Of note, IELPs from *Id3*^*Gfp/Gfp*^ mice showed a significant increase in GFP expression when compared to *Id3*^*Gfp/+*^ mice (Fig. 2a, b). One potential explanation for the higher GFP levels in *Id3*^*Gfp/Gfp*^ mice could be that GFP is expressed from both *Id3* alleles. Hence, this would translate into a doubling of GFP expression, if it were the only reason. In addition, the absence of T-bet^+^ IELPs in the thymus of *Id3*^*Gfp/Gfp*^ mice could generate a backward loop that provides *Id3*-inducing signals to compensate for that loss, which would also lead to increase in GFP in *Id3*^*Gfp/Gfp*^ mice. To get more insight, we analyzed IELPs from *Id3*^*Gfp/+*^ and *Id3*^*Gfp/Gfp*^ mice with additional markers versus GFP. For example, plotting GFP versus the developmental markers CD24 (decreases during maturation, Klose et al.^15^) allowed us to distinguish three major populations (i.e., CD24^+^GFP^−^; CD24^+^GFP^+^; CD24^−^GFP^+^), of which two were GFP^+^ (Fig. 2d, e). Comparing the mean fluorescent intensity (MFI) for GFP in the corresponding subpopulation showed an increase by a factor of >4 in CD24^+^GFP^+^ but only a factor of 2 in more mature CD24^−^GFP^+^ IELPs (Fig. 2e). This would favor the idea of a bi-allelic *Id3* (*Gfp*) expression in combination with *Id3*-inducing signals in at least a subpopulation of IELPs from *Id3*^*Gfp/Gfp*^ mice. Despite this potential bi-allelic expression, *Id3*^*Gfp/+*^ mice showed no overt phenotype and no perturbances in their IELPs or CD8α^+^ IELs. Similar to Id3, Nur77 is induced and regulated by the strength of the TCR signal during thymic development. Interestingly, we saw a significant drop in the percentage of Nur77 in IELPs from *Id3*^*Gfp/Gfp*^ mice (Fig. 2f, g). Hence, our data would be in line with a model in which absence of Id3 leads to perturbed TCR signaling cascade, demonstrated by a reduction in Nur77 expression, that impedes clonal deviation into the IEL lineage. In contrast to the results from the reporter mice, quantitative real-time PCR (qPCR) from sorted IELP subpopulations showed a profound reduction in *Id3* mRNA in CD122^+^T-bet^−^ and CD122^+^T-bet^+^ IELPs (Supplementary Fig. 2b), which is also in line with our scRNA-seq data. This discrepancy between *Id3* mRNA and Id3-GFP expression was not restricted to IELPs but also observed in other thymocyte populations (Supplementary Fig. 2a, b) and has been shown before.^30^ Hence, it is most likely that the expression or stability of GFP is differentially regulated in different thymocyte subsets and not always fully reflects endogenous *Id3* expression in wild-type (WT) mice. This must be taken into account when working with Id3 reporter mice. Ultimately, Id3 deficiency resulted in a significantly reduced numbers of CD8α^+^ TCRαβ^+^ IELs in the small intestine of *Id3*^*Gfp/Gfp*^ mice, whereas CD8αβ^+^ TCRαβ^+^ IELs were overrepresented as compared to *Id3*^*Gfp/+*^ mice (Fig. 2h, i). CD8αα^+^ TCRγδ^+^ IELs were not affected by the absence of Id3 (Fig. 2h, i, Supplementary Fig. 2c). The observed reduction of *Id3* during IELP differentiation (Supplementary Fig. 2b) continued in the small intestine, where the majority of CD8α^+^ TCRαβ^+^ IELs had lost GFP, whereas the CD8αβ^+^ TCRαβ^+^ IELs showed a bimodal pattern similar to conventional memory T cells (Fig. 2j). The fact that CD8αα^+^ TCRαβ^+^ IELs are T-bet^+^ and CD122^+^ but Id3^−^ (GFP^−^) in *Id3*^*Gfp/+*^ mice (Fig. 2j) clearly shows that Id3 is not essential for the maintenance of T-bet or CD122 in WT mice. In summary, this data is consistent with a model in which the timely expression of Id3 during thymic development is important for the induction of CD122 and T-bet in IELPs, which cannot be fully compensated for in *Id3*^*Gfp/Gfp*^ mice.

The continuous downregulation of Id3 was reminiscent of T-bet^+^ iNKT1, which similar to CD8αα^+^ IELs also depend on IL-15 and T-bet for their development. This analogy was further strengthened by the continuous and inverse increase of Id2 as shown in the scRNA-seq data (Fig. 1c) and corroborated by reverse transcription (RT)–qPCR of sorted thymocytes populations (Fig. 2k). Finally, analysis of Id2 reporter mice (*Id2*^*Gfp/+*^) confirmed continuous increase of Id2 expression, by demonstrating a continuous increase of GFP (Id2) during thymic maturation of IELPs (Fig. 2l) and the highest GFP expression in intestinal CD8αα^+^ IELs (Fig. 2m, n). Hence, we can conclude that CD8αα^+^ IELs and iNKT1 share important components of their transcriptional circuitry, beyond T-bet, that also involves Id proteins.

### C-Myc regulates the induction of T-bet in unconventional and conventional T cells after TCR simulation

PD-1 is a temporary marker for thymocytes that have received a strong TCR stimulation and it has been suggested that these cells are destined for clonal deletion (conventional T cells) or clonal diversion (unconventional T cells, e.g., natural IELs, NKT cells).^4,12^ Although natural IELs require a strong agonist signal during their thymic development, there have been conflicting data in the past, whether all natural TCRαβ^+^ IELs progress through a PD-1^+^ stage during thymic development.^10,13^ In the thymus of WT (not shown) and TBGR mice, a large proportion of NK1.1^−^ IELPs are expressing PD-1, which is gradually lost upon the expression of T-bet (Supplementary Fig. 3a), and as a result T-bet^high^ cells are PD-1^−^. This is well in line with our scRNA-seq data (Fig. 1g). Hence, natural IELs, as well as induced IELs, are negative for PD-1 while expressing high levels of T-bet (Supplementary Fig. 3b). A similar waterfall-shaped profile for PD-1 and T-bet was also observed in agonist-selected NKT cells during thymic development (Supplementary Fig. 3c).

**Fig. 3:**
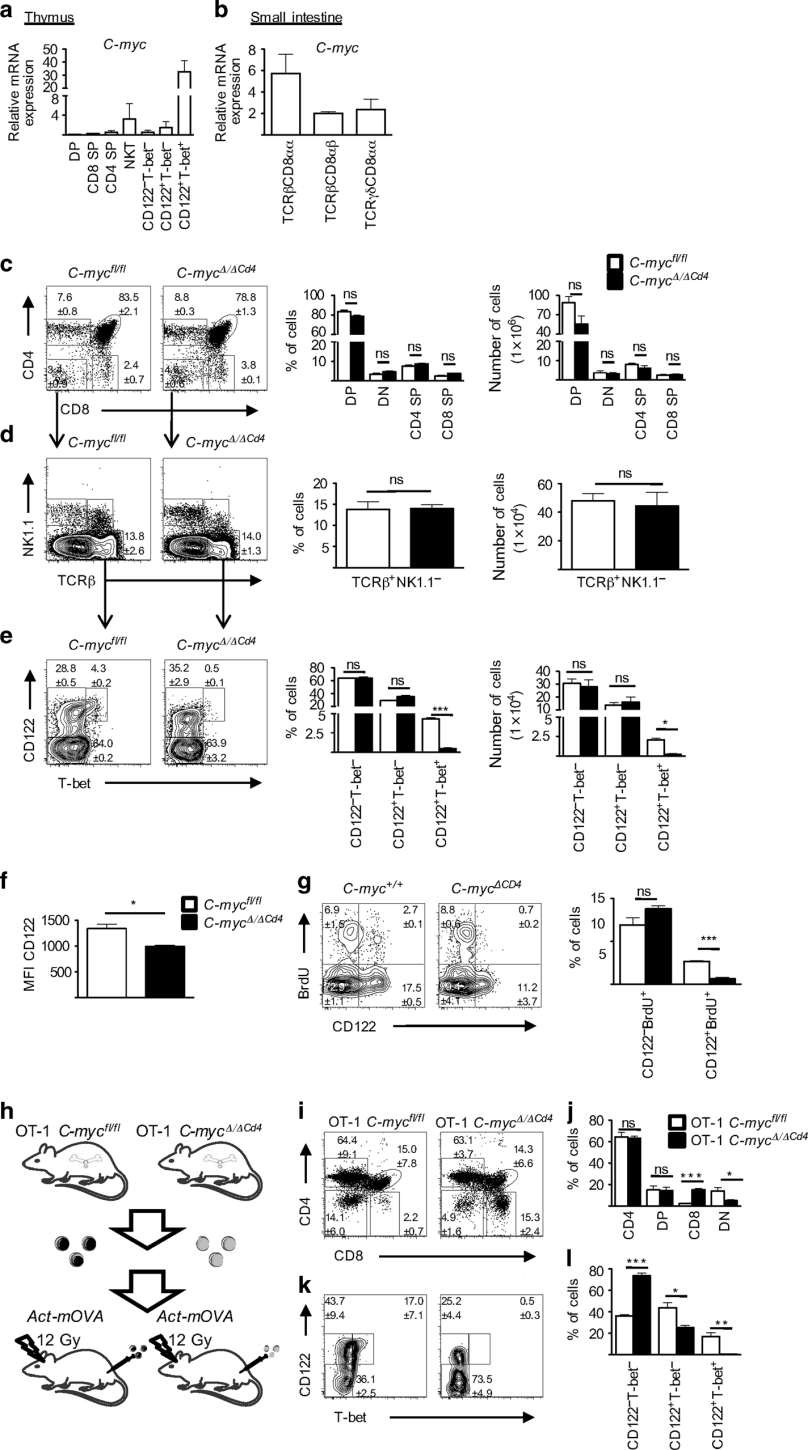
C-myc regulates the induction of T-bet in unconventional T cells after TCR simulation. **a** Bar diagram shows *C-myc* mRNA expression (Mean ± SEM) of different thymocytes population from three 6–12-week-old TBGR mice performed by qPCR. Pre-gating is shown in Supplementary Fig. 1a. **b** *C-myc* mRNA expression for natural (TCRαβ^+^ or TCRγδ^+^ CD8αα^+^) and induced (TCRαβ^+^ CD8αβ^+^) IELs isolated from the small intestine of three 6–12-week-old TBGR mice and analyzed by qPCR. IELs were pre-gated for DAPI^−^CD45^+^CD4^−^. **c** Dot plot shows flow cytometric analysis of thymocyte subsets from 6- to 12-week-old *C-myc*^Δ/Δ*Cd4*^ mice and littermate *C-myc*^*fl/fl*^ mice as controls. Further flow cytometric analysis of DN thymocytes (**d**) and NK1.1^−^ IELPs (**e**) as in **c**. **c**–**e** Bar diagrams represent percentages (left, Mean ± SEM) and absolute cells numbers (right, Mean ± SEM) of the respective thymocyte populations. **f** Histogram shows MFI of CD122 in thymic NK1.1^−^CD122^+^T-bet^−^ IELPs as shown in **e**. **g** Flow cytometric analysis of BrdU incorporation in NK1.1^−^ IELPs after 3 h of BrdU pulse labeling (contour plots) and bar diagram shows statistical analysis (Mean ± SEM) of BrdU^+^ subsets (right panel) of 2-week-old *C-myc*^Δ/Δ*Cd4*^ mice and littermate *C-myc*^*fl/fl*^ mice as controls for 5 mice per genotype. **h** Schematic model for the generation of bone marrow chimeras. **i** Representative dot plot shows flow cytometric analysis of different thymocyte populations from five OT-1 *C-myc*^*Δ/ΔCd4*^ bone marrow chimeras and *C-myc*^*fl/fll*^ littermate controls. Cells were pre-gated on TCR Vα2 and numbers denote the percentage of cells in the gate (Mean ± SEM) of five mice per group. **k** Further flow cytometric analysis as in **h** for NK1.1^−^ IELPs classified upon their CD122 and T-bet expression. **j**, **l** Bar diagrams show the percentage (Mean ± SEM) of the respective thymocyte subpopulation

Having shown that PD-1 precedes the expression of T-bet in NK1.1^−^ IELPs, we wanted to investigate how this transition is regulated in IELPs. Although it has been shown that TCR signaling and IL-15 are both important for T-bet induction,^14^ it has remained elusive how these signals are integrated at a transcriptional level. One potential candidate was C-Myc. TCR activation and cytokine signaling cooperatively induce C-Myc,^16^ and it has been demonstrated that conditional knockout mice for C-Myc lack natural TCRαβ^+^ IELs,^19^ without affecting their thymic precursors. Investigating *C-Myc* expression along our inferred trajectory showed the expression onset at the CD122^+^T-bet^−^ stage, which was maintained at low levels throughout the CD122^+^T-bet^int^ stage (Supplementary Fig. 3d). Based on this low expression, we initially did not predict it as a relevant target. In contrast, transcriptional expression analysis of distinct IELP stages and other T cell lineages in TBGR mice showed a striking positive correlation between *C-myc* and *Tbx21* in the thymus (Fig. 3a). Intriguingly, this correlation was not maintained in the intestine (Fig. 3b). To gauge the hierarchy between these two factors, we analyzed and compared conditional T cell-restricted C-Myc knockout mice (*Cd4*^*Cre-*^^*Tg*^;*C-myc*^*fl/fl*^ hereafter called *C-myc*^*Δ/ΔCd4*^) with the relevant littermate controls (*C-myc*^*fl/fl*^). The absence of C-Myc in T cells had no effect on the absolute or relative numbers of DN, DP, CD4 SP, and CD8 SP thymocytes (Fig. 3c). In line with previous results,^19^ we did not detect any difference in the number of NK1.1^−^ IELPs (Fig. 3d), whereas NKT cells were reduced. However, careful analysis of NK1.1^−^ IELPs clearly demonstrated that *C-myc*^*Δ/ΔCd4*^ mice had strikingly reduced CD122^+^T-bet^+^ IELPs (Fig. 3e) and a striking reduction of TCRαβ^+^ CD8αα^+^ IELPs in the small intestine (Supplementary Fig. 3e). Partially overlapping with the phenotype of *Id3*^*Gfp/Gfp*^ mice, we detected a significant, yet less pronounced, decrease in the MFI of CD122 in CD122^+^T-bet^−^ IELPs (Fig. 3e, f), which implies that C-Myc regulates the expression of CD122 independent of T-bet at that stage. C-Myc is a well-known regulator of cell growth and proliferation, and therefore we investigated its role in IELPs using 5-bromo-2’-deoxyuridine (BrdU) pulse-labeling studies. Here we observed a significant reduction of BrdU incorporation in CD122^+^ IELPs but not in CD122^−^ IELPs (Fig. 3g). Hence, the effects of C-Myc deficiency might in part be explained by its role in cell proliferation during later stages; however, this does not necessarily explains the lack of T-bet in IELPs. In conclusion, the pronounced upregulation of C-Myc during thymic IELP development is essential for the induction of T-bet, which in turn regulates proliferation and differentiation of IELs in response to IL-15.^14^

So far, our data have interrogated the role of C-Myc in a polyclonal TCR repertoire. To follow the fate of individual T cells in WT and C-Myc knockouts, we crossed *C-myc*^*Δ/ΔCd4*^ mice with OT-1 TCR transgenic mice that harbor a transgenic TCR specific for SIINFEKL restricted by H2-K^b^. Next, we created two groups of bone marrow chimeras by lethally irradiating Act-mOVA mice and reconstituting them with bone marrow from either OT-1 C-Myc sufficient (OT-1 *C-myc*^*fl/fl*^) or OT-1 C-Myc deficient (OT-1 *Cd4*^*Cre-Tg*^;*C-myc*^*fl/fl*^, hereafter called OT-1 *C-myc*^*Δ/ΔCd4*^) mice (Fig. 3h). Transfer of OT-1 *C-myc*^*fl/fl*^ cells leads to thymic high-avidity interaction between the TCR and the ubiquitously presented SIINFEKL peptide that is derived from Ovalbumin. This thymic interaction results in wide-spread clonal deletion of thymocytes expressing the transgenic TCR (Vα2^+^), which therefore fail to reach the CD8 SP stage, (Fig. 3i (left panel) and Fig. 3j), and concomitant clonal diversion into the IEL lineage with pronounced expression of T-bet^+^ (Fig. 3k (left panel) and Fig. 3l).^14^ In contrast, Vα2^+^ OT-1 T cells from OT-1 *C-myc*^*Δ/ΔCd4*^ mice showed significantly more CD8 SP T cells and reduced numbers of IELPs (Fig. 3i (right panel) and Fig. 3j). Similar to polyclonal T cells there was no induction of T-bet (Fig. 3k (right panel) and Fig. 3l). We also detected an accumulation of CD122^−^ T-bet^−^ IELPs in Vα2^+^ OT-1 T cells from OT-1 *C-myc*^*Δ/ΔCd4*^ mice (Fig. 3k (right panel) and Fig. 3l) that was not evident in *C-myc*^*Δ/ΔCd4*^ mice with a polyclonal repertoire (Fig. 3e), which is probably the result of the TCR transgenic system used. Hence, we can conclude that even high-avidity interaction in a restricted TCR repertoire cannot compensate for the lack of C-Myc in thymic IEL development. Furthermore, these experiments also suggest, that not only clonal diversion but also clonal deletion is influenced by C-Myc due to the emergence of CD8 SP Vα2^+^ OT-1 T cells in OT-1 *C-myc*^*Δ/ΔCd4*^ → Act-mOVA chimeras (Fig. 3i, j).

**Fig. 4:**
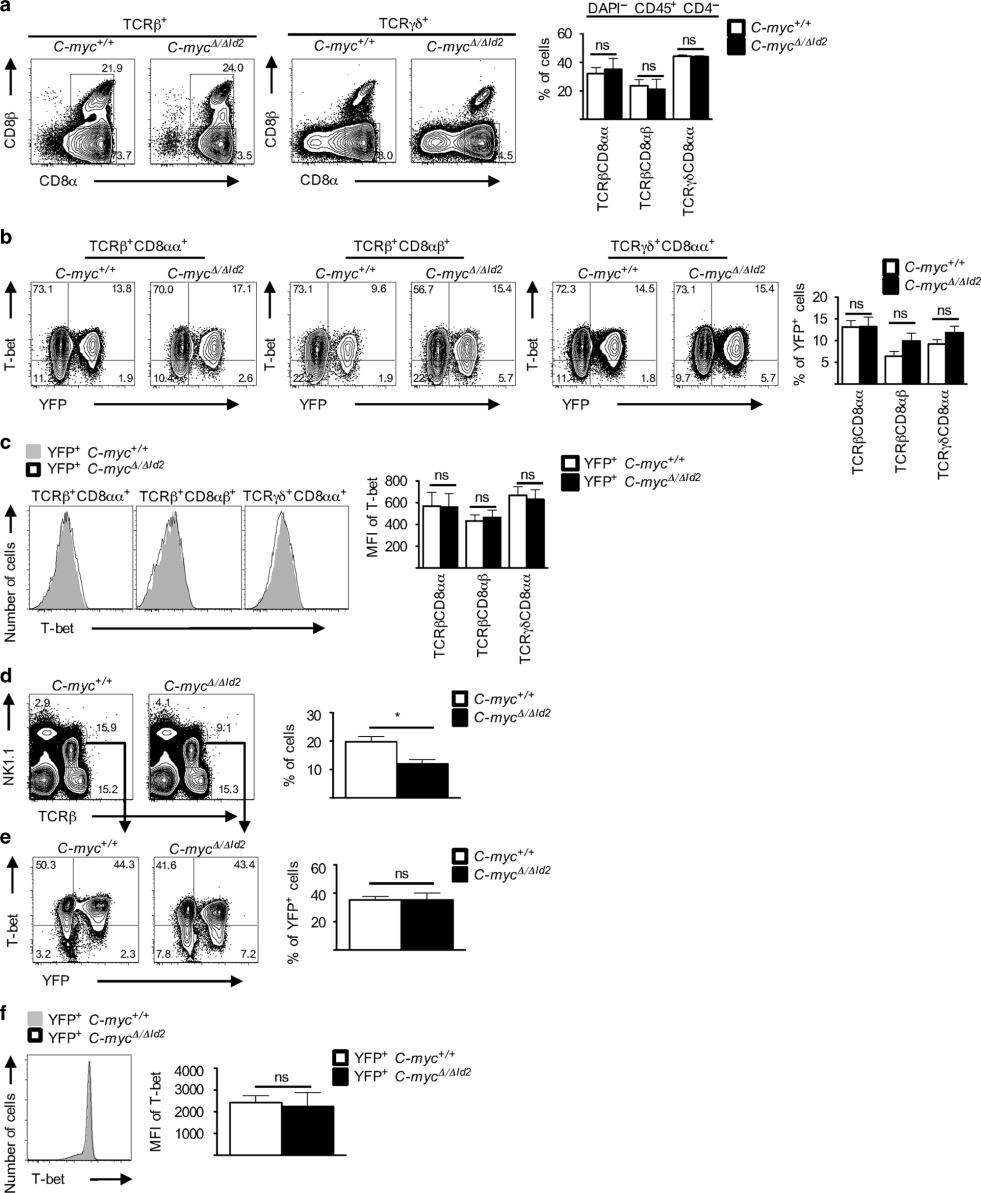
C-myc regulates the induction of T-bet in unconventional T cells. **a** Flow cytometric analysis of freshly isolated lymphocytes isolated from the epithelium of the small intestine from 4- to 5-month-old *C-myc*^Δ/Δ*Id2*^ mice and as controls *C-myc*^*+/+*^ littermate mice after 3 weeks of tamoxifen treatment. Dot plot is pre-gated on DAPI^−^ CD45^+^ CD4^−^ and analyzed for TCRβ^+^ or TCRγδ^+^—as indicated—and bar diagram shows the percentages (Mean ± SEM) of at least eight mice per genotype. **b** Further analysis of co-expression of YFP and T-bet in IEL subsets. Bar diagram shows the percentages of YFP^+^ cells (Mean ± SEM). **c** Histograms represent T-bet expression of TCRβ^+^ and TCRγδ^+^ IELs and bar diagram shows MFI of T-bet expression (Mean ± SEM). **d** Flow cytometric analysis of hepatic lymphocytes of 3–5-month-old *C-myc*^Δ/Δ*Id2*^ mice and *C-myc*^*+/+*^ littermate controls after 3 weeks of tamoxifen treatment. Bar diagram (right) shows the percentages of hepatic NKT cells (Mean ± SEM) of at least four mice per genotype. **e** Further flow cytometric analysis of hepatic NKT cells (TCRβ^+^ NK1.1^+^) for YFP and T-bet expression. Bar diagrams (right) show the percentages of YFP^+^ cells (Mean ± SEM). **f** Overlay histogram (left) shows representative T-bet expression of hepatic NKT cells (gated as in **d**, **e**) and bar diagram shows MFI of T-bet expression (Mean ± SEM)

Of note, the strong positive correlation between T-bet and C-Myc was lost in the periphery (Fig. 3b), where it was potentially less relevant. To investigate this directly, we generated inducible conditional *C-myc* knockout mice by crossing *Id2*^*CreERT2/+*^ with *C-myc*^*fl/fl*^ (*Id2*^*CreERT2/+*^;*C-myc*^*fl/fl*^). Id2 is highly expressed in IELs (Fig. 2m, n) and tamoxifen treatment translocates the CreERT2 into the nucleus, where it induces recombination to generate a functional *C-myc* null allele. To track cells with translocated, CreERT2 mice were further bred with Rosa26-reporter mice (*Id2*^*CreERT2/+*^;*C-myc*^*fl/fl*^;*Rosa26R*^*Yfp/+*^). We next treated *Id2*^*CreERT2/+*^;*C-myc*^*fl/fl*^;*Rosa26R*^*Yfp/+*^ (hereafter called *C-myc*^*Δ/ΔId2*^) and *Id2*^*CreERT2/+*^;*C-myc*^*+/+*^;*Rosa26R*^*Yfp/+*^ (hereafter called *C-myc*^*+/+*^) control mice for 3 weeks with a tamoxifen-containing diet and analyzed their IEL compartment in the small intestine. Genomic PCR of sorted populations confirmed that yellow fluorescent protein (YFP) is a suitable surrogate for efficient recombination at the C-myc locus in *C-myc*^*Δ/ΔId2*^ mice (Supplementary Fig. 3f). There was no difference in the numbers and distribution of IEL subsets in *C-myc*^*+/+*^ and *C-myc*^*Δ/ΔId2*^ mice (Fig. 4a, b). Moreover, and in accordance with our transcriptional data (Fig. 3b), there was no loss of T-bet expression in IELs after conditional deletion of *C-myc* and these cells remained viable during the course of the experiment (Fig. 4c). Hence, C-Myc is essential for induction but not maintenance of T-bet during IEL development. Similar results for C-Myc ablation were shown in liver-resident NKT cells, where, despite slightly reduced absolute numbers (Fig. 4d), the inducible loss of C-Myc neither affected the frequency of YFP^+^ NKT cells nor the expression of T-bet (Fig. 4e, f).

### C-Myc-deficient IELPs show developmental arrest after agonist selection

**Fig. 5:**
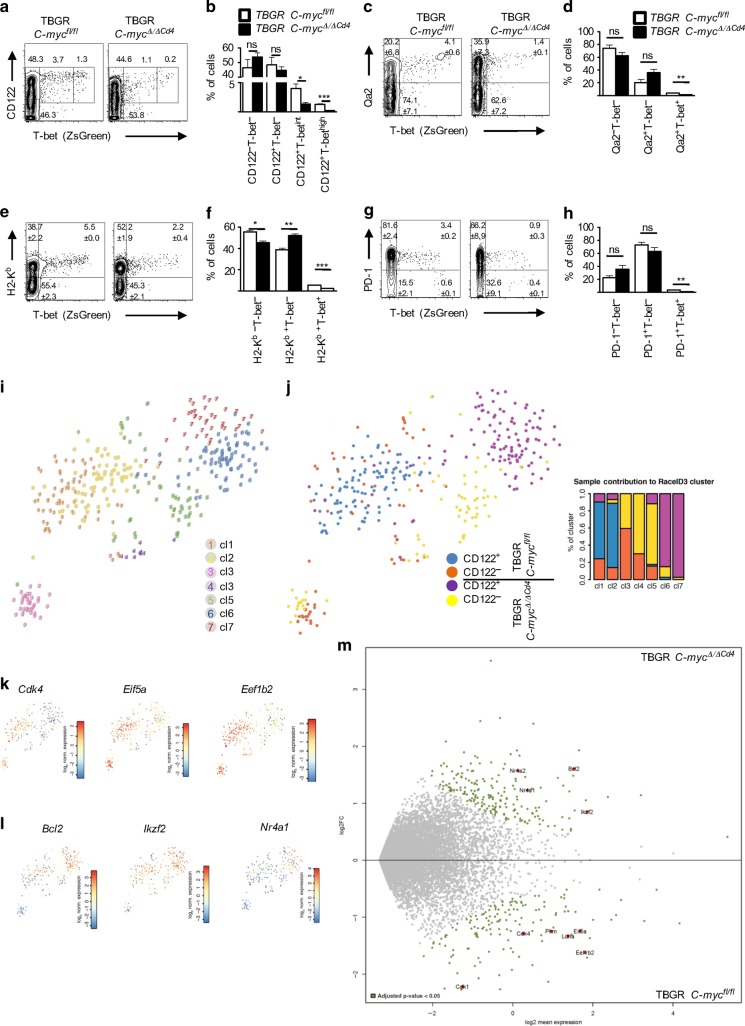
C-myc knockout NK1.1^−^ IELPs show developmental arrest after agonist selection. **a**–**h** Flow cytometric analysis of thymic NK1.1^−^ IELPs from 6- to 16-week-old TBGR *C-myc*^*Δ/ΔCd4*^ mice and TBGR *C-myc*^*fl/fl*^ littermate mice as controls. Dot plot shows T-bet vs. CD122 (**a**), Qa2 (**c**), H2-K^b^ (**e**), and PD-1 expression (**g**). Numbers denote the percentage of cells in the gate (Mean ± SEM) for at least six mice per genotype. Bar diagram shows statistical analysis of (Mean ± SEM) T-bet vs. CD122 (**b**), Qa2 (**d**), H2-K^b^ (**f**), and PD-1 expression (**h**) in NK1.1^−^ IELPs. **i** t-SNE map representing RaceID3 clustered cells from TBGR *C-myc*^*Δ/ΔCd4*^ and TBGR *C-myc*^*fl/fl*^ littermate mouse as controls. Note that owing to the absence of T-bet^+^ IELPs in *C-myc*^*Δ/ΔCd4*^ mice only T-bet^−^ cells are analyzed and shown for these samples. **j** t-SNE map showing the sample information of RaceID3 clustered cells. Bar diagram shows the relative contribution of individual samples to the seven different clusters identified by RaceID3. **k**, **l** t-SNE representation of the selected genes corresponding to relevant markers upregulated in T-bet^−^ TBGR *C-myc*^*fl/fl*^ (*Cdk4*, *Eif5a*, and *Eef1b2* (**k**)) or T-bet^−^ TBGR *C-myc*^*Δ/ΔCd4*^ (*Bcl2*, *Ikzf2*, and *Nr4a1* (**l**)) IELPs within the different clusters as shown in **i**. **m** MA plot of differential expression analysis between CD122^+^T-bet^−^ cells from TBGR *C-myc*^*Δ/ΔCd4*^ mice (top) versus CD122^+^T-bet^−^ cells from age-matched (2–3 weeks old) TBGR *C-myc*^*fl/fl*^ control mice (bottom). Differentially expressed genes involved in agonist selection, translational initiation, and C-Myc target genes involved in cell-cycle regulation and energy metabolism are highlighted. The ensemble of all *Rps*, *Rpl*, *Rik*, *Gm*, and *RP* genes are not displayed in the MA plot; please refer to Supplementary Fig. 4e. Bonferroni adjusted *p* value < 0.05

To better understand the phenotypic consequences of C-Myc deficiency in IEL development, we crossed C-Myc-deficient mice (*Cd4*^*Cre-Tg*^;*C-myc*^*fl/fl*^) with TBGR mice (hereafter caller TBGR *C-myc*^*Δ/ΔCd4*^). The superior brightness and dynamic range of ZsGreen allows the detection of even subtle changes in *Tbx21* expression and can be used for cell sorting based on ZsGreen expression (Fig. 1). As predicted from Fig. 3c, TBGR *C-myc*^*Δ/ΔCd4*^ mice showed no difference in absolute or relative numbers of DN, DP, CD4 SP, and CD8 SP thymocytes, whereas thymic NKT cells were absent (Supplementary Fig. 4a, b). Furthermore, there was a significant decrease in T-bet^int^ and T-bet^high^ NK1.1^−^ IELPs (Fig. 5a, b). Absence of C-Myc had no effect on the acquisition of early maturation markers like Qa2 (Fig. 5c, d) or H2-K^b^ (MHC class I) (Fig. 5e, f). Moreover, PD-1 expression was still present (Fig. 5g, h), arguing that C-Myc has no relevant effect on PD-1 but indeed regulates the transition from PD-1^+^T-bet^−^ to PD-1^−^T-bet^high^ state in thymic NK1.1^−^ IELPs.

Having shown that C-Myc is required for the induction of T-bet and thus the commitment of IELPs, we wanted to investigate at which stage the block in IELP differentiation occurs. As T-bet^+^ IELPs are almost absent in the thymus of TBGR *C-myc*^*Δ/ΔCd4*^ mice, we sorted CD122^−^T-bet^−^ and CD122^+^T-bet^−^ NK1.1^−^ IELPs from 2- to 3-week-old TBGR *C-myc*^*Δ/ΔCd4*^ mice together with the corresponding CD122^−^T-bet^−^ and CD122^+^T-bet^−^ NK1.1^−^ IELPs from age-matched TBGR *C-myc*^*fl/fl*^ (WT) mice as in the initial scRNA-seq experiment (Supplementary Fig. 1a). αGalCer:CD1d^+^ tetramer staining of thymocytes from TBGR *C-myc*^*Δ/ΔCd4*^ mice demonstrated the severe reduction of NKT cells (Supplementary Fig. 4c), which were virtually absent in sorted DN TCRαβ^+^NK1.1^−^ IELPs (Supplementary Fig. 4d). After library preparation and sequencing, we obtained 347 high-quality RNA profiles of IELPs with at least 2000 transcripts per cell, which were used as input for clustering using RaceID3. In total, we obtained 8 clusters, of which 7 had at least 10 cells (Fig. 5i). At the early CD122^−^ stage, IELPs from TBGR *C-myc*^*fl/fl*^ and TBGR *C-myc*^*Δ/ΔCd4*^ mice clustered jointly in clusters 3 and 4 (Fig. 5j, t-SNE plot and bar diagram). In contrast, CD122^+^ IELPs from TBGR *C-myc*^*Δ/ΔCd4*^ mice clustered separately from their WT counterparts in clusters 6 and 7, whereas the majority of CD122^+^ cells from WT mice (TBGR *C-myc*^*fl/f*^^*l*^) clustered in RaceID3 clusters 1 and 2 (Fig. 5j).

CD122^+^ WT cells, largely comprising clusters 1 and 2, express higher levels of the s-ribosomal and l-ribosomal gene family genes (Supplementary Fig. 4e); translational elongation factors, such as eukaryotic elongation factor 1 beta 2 (*Eef1b2*) and eukaryotic translation initiation factor 5A (*Eif5a*); and the cyclin-dependent kinases *Cdk1* and *Cdk4* (Fig. 5k, m). Conversely, CD122^+^T-bet^−^ cells from TBGR *C-myc*^*Δ/ΔCd4*^ mice within clusters 6 and 7 express lower levels of these genes (Fig. 5k). Instead, they exhibit upregulation of genes associated with agonist selection such as *Ikzf2*, *Nr4a1*, and *Bcl2* (Fig. 5l and Supplementary Fig. 4e). Interestingly, in our inferred trajectory in Fig. 1a the expression of the known C-Myc targets *Eif5a* and *Cdk4*^31,32^ peaks in the CD122^+^T-bet^−^ stage and declines at the transition to the CD122^+^T-bet^int^ stage (Supplementary Fig. 4f), reminiscent of the expression of *Ikzf2* at this transition (Fig. 1d). Finally, and in line with C-Myc’s role in energy metabolism of activated T cells,^18^ CD122^+^ IELPs from *C-myc*^*Δ/ΔCd4*^ mice also showed a significant decrease in key enzymes of glycolysis, such as lactate dehydrogenase A (*Ldha*) and muscle type of pyruvate kinase (*Pkm*) (Fig. 5m). In summary, the absence of C-Myc, besides its effects on the cell cycle and energy supply, leads to an impairment of the translational machinery through—direct or indirect—downregulation of ribosomal genes and genes involved in translational elongation. Furthermore, our data suggest that this results in a block in IELP differentiation during or shortly after agonist selection.

## Discussion

**Fig. 6:**
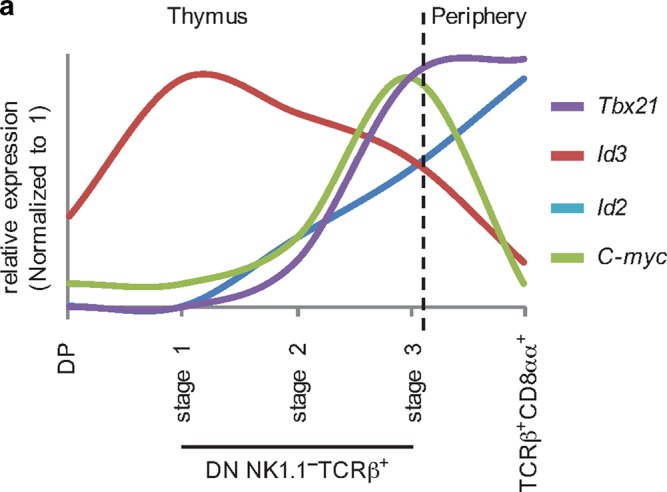
Schematic summary of the relative expression of transcriptional regulators during TCRαβ^+^ CD8αα^+^ IEL differentiation. Scheme shows the relative expression (normalized to 1) of *Tbx21* (purple), *Id2* (blue), *Id3* (red), and *C-myc* (green) along different stages during IEL development derived from the presented qPCR and scRNA-seq data. Thymus: DP: CD4^+^CD8^+^, stage 1: CD4^−^CD8^−^TCRβ^+^NK1.1^−^CD122^−^T-bet^−^, stage 2: CD4^−^CD8^−^TCRβ^+^NK1.1^−^CD122^+^T-bet^−^, stage 3: CD4^−^CD8^−^TCRβ^+^NK1.1^−^1CD22^+^T-bet^+^

In this study, we used scRNA-seq in combination with in vivo models to define and validate a linear thymic IELP differentiation model of NK1.1^−^ IELPs that further strengthen the notion that these cells are selected through high-avidity interactions. As a result, NK1.1^−^ IELPs showed an early expression of Helios and Nur77 after positive selection and progress through a PD-1^+^ stage before gradually inducing T-bet in a C-Myc-dependent manner. Moreover, we identified Id3, and possibly Id2, as novel regulators of thymic IELP development with significantly reduced numbers of CD122^+^T-bet^−^ and CD122^+^T-bet^+^ IELPs in *Id3*^*Gfp/Gfp*^ mice (Fig. 6).

Id3 is strongly upregulated upon pre-TCR and TCR signaling during thymocyte development and progressively increases until T cells reach the SP stage.^28,29^ During this passage, Id3 opposes the actions of E proteins, which are important during pre-β-selection steps by activating TCRβ rearrangement, inhibiting proliferation, and supporting Notch signaling as well as pre-TCR signaling.^29,33^ In line with that, TCR transgenic mice on an Id3-deficient background show defects in positive and negative selection. However, whether this also takes place in a polyclonal repertoire is still a matter of debate as deletion of T cells specific for endogenous superantigens is unperturbed in Id3-deficient mice. Intriguingly, overexpression of Id3 in a murine DP cell line mimics IELP development by upregulation of TCRαβ, CD69, CD5, and CD44 and, more important, downregulation of CD24 (HSA), CD4, and CD8.^34^ This gain-of-function model is well in accordance with our loss-of-function model (*Id3*^*Gfp/Gfp*^), which places Id3 before IELPs express CD122, and therefore upstream of T-bet. Transforming growth factor (TGF)-β signaling can induce Id3 in B cells^35^ and, vice versa, constitutive or conditional *Tgfb1* knockouts show a significant reduction of IELPs and CD8αα^+^ TCRαβ IELs but not CD8αα^+^ TCRγδ IELs.^36^ Similarly, CD8αα^+^ TCRγδ IELs also showed no reduction in *Id3*^*Gfp/Gfp*^ mice. Whether like-wise interactions between TGF-β and Id3 also take place during IELP development is currently unclear. It is important to stress that the phenotype of *Id3*-deficient mice is complex as it affects various T cell lineages via intrinsic and extrinsic mechanisms. For example, thymi from Id3-deficient mice harbor innate-like CD8 T cells that constitutively express CD122, CD44, and Eomes and are potent producers of interferon-γ. Intriguingly, their thymic generation is extrinsically regulated by PLZF^+^ iNKT cells, which produce high levels of IL-4. However, this effect on CD122 expression is not observed in IELPs from *Id3*^*Gfp/Gfp*^ mice, which rather show significant reduction of CD122^+^ IELPs. In addition, naive CD8 T cells, which lack CD122 expression, are uniformly Id3^+^ (Supplementary Fig. 2a). In summary, there is no clear correlation between the expression of CD122 and Id3. Hence, it is unlikely that the reduction of IELPs in *Id3*^*Gfp/Gfp*^ mice mediated through a direct effect of Id3 on CD122 expression levels. iNKT cells, especially T-bet^+^ iNKT1 cells, share many developmental features with CD8αα^+^ IELs, like their dependency on IL-15 and T-bet. Moreover, we show here that this also extends to Id2 and Id3, where the latter is essential for T-bet^+^ iNKT1. *Id3* expression declines after lineage specification, whereas *Id2* shows a continuous increase during IEL and iNKT development. This might also explain the discrepancy between the striking effects of Id3 deficiency on thymic IELPs and the milder phenotype in the intestine of *Id3*^*Gfp/Gfp*^ mice, where Id2 might be able to partially compensate for the loss of Id3. Finally, the lack of expression of Id3 in CD8αα^+^ IELs clearly indicates that Id3 does not play a role in maintenance of CD8αα^+^ IELs but rather serves as checkpoint at an early thymic stage that allows progression into the IEL lineage. In summary, the presented data clearly establishes an important role for Id3 in IEL development but further studies will be needed to delineate its specific step-wise actions and potential redundancy in the presence of Id2.

Recent advances in scRNA-seq and data analysis have been harnessed to identify cell types as well as precursor–progeny relationships in complex biological samples, which were previously not accessible.^24,25^ Applying scRNA-seq on the heterogeneous population of NK1.1^−^ IELPs allowed us in an unbiased workflow to untangle this heterogeneity and define the developmental trajectory of NK1.1^−^ IELPs in the thymus. Inference of differentiation dynamics clearly illustrated that CD122^−^T-bet^−^ (cluster 5, Fig. 1) and CD122^+^T-bet^+^ (cluster 2, Fig. 1a) IELPs are at opposing ends of that developmental process. Moreover, the fact that the five described clusters form a single trajectory with no branching points also implicates that NK1.1^−^T-bet^high^ IELPs arise from a common thymic precursor. To further corroborate this finding, we next mined the data for specific marker genes and singled out PD-1, as it was early expressed in cluster 3 and clearly before T-bet. This would support a model in which all NK1.1^−^T-bet^high^ IELPs progress through a PD-1 stage before they upregulate T-bet. Furthermore, a human study also identified PD-1^+^ T cells in the thymus and cord blood as agonist-selected T cells that expressed CD8αα.^37^ This is also in line with elegant studies which demonstrated that the selective and timely expression of a large range of TCRs from individual CD8αα^+^ TCRαβ^+^ IELs in developing thymocytes lead to the induction of PD-1 and the exclusive development of CD8αα^+^ TCRαβ^+^ IELs.^10,11^ Intriguingly, a recent report suggested that CD8αα^+^ TCRαβ^+^ IELs have a dual thymic origin, with only a proportion of agonist-selected T cells.^13^ This study used a different T-bet reporter mouse strain (*Tbx21*^*Gfp*^) and identified IELPs as DN CD25^−^CD1dtet^−^CD5^+^TCRβ^+^ thymocytes (excluding regulatory T cells and invariant NKT cells), which were further subdivided into two main populations: PD-1^+^NK1.1^−^T-bet^−^ versus PD-1^−^NK1.1^+^T-bet^high^. Most importantly, the authors claimed that these two populations were not related in a precursor–progeny relationship and both gave rise to CD8αα^+^ TCRαβ^+^ IELs after adoptive transfer.^13^ In accordance with our current study, the authors also demonstrated that NK1.1^−^ IELPs, which express PD-1, are the result of strong agonist interactions during thymic development and have a largely immature phenotype with respect to CD24, H2-K^b^, Qa-2, and CD122. However, there are also important aspects that were not addressed in the study by Ruscher et al.^13^ Foremost, NK1.1^−^ IELPs contain a significant proportion of cells that express T-bet, whose expression correlates with maturation.^14,15^ Here we show through scRNA-seq that these cells are developmentally related to T-bet^−^ NK1.1^−^ IELPs and that their differentiation non-redundantly depends on the expression of C-myc. It stands to reason that NK1.1^+^ IELPs also depend on C-Myc as conditional *C-myc* knockout mice are lacking all CD8αα^+^ TCRαβ^+^ IELs. Interestingly, our scRNA-seq data show a *Klrb1c* (encodes for NK1.1) signature in T-bet^+^ NK1.1^−^ IELPs (cluster 2, Supplementary Fig. 1c) and as a result NK1.1 can be detected on CD8αα^+^ TCRαβ^+^ IELs, which further underlines the difficulties in distinguishing the intestinal progeny of thymic “type A” and “type B” precursors.^13^

Finally, we wanted to understand upstream regulators of T-bet in IEL development. C-Myc was a suitable candidate, as *C-myc*^*Δ/ΔCd4*^ mice lack CD8αα^+^ TCRαβ^+^ IELs.^19^ C-Myc is known to integrate signals from the TCR and cytokine receptors.^16^ This would be in agreement with our previous report which showed that the combined actions of TCR stimulation and IL-15 signaling regulate the induction of T-bet in IELPs.^14^ Analysis of *C-myc*^*Δ/ΔCd4*^ mice supported this hypothesis as these mice were devoid of T-bet^+^ IELPs and CD8αα^+^ TCRαβ^+^ IELs, while PD1^+^T-bet^−^ IELPs were still present. In conventional T cells, strong TCR activation rapidly induces C-Myc, which is then maintained by IL-2 or IL-15 and this is crucial for T cell activation as failure of C-Myc expression is associated with a defect in T cell activation.^16,38^ This early induction is necessary for T cell growth and proliferation,^18^ which are both hampered in *C-myc*^*Δ/ΔCd4*^ mice. It is therefore not surprising that C-Myc affects IEL development, which shares many features with activated or memory T cells.

Although we detected some effects of *C-myc* deficiency on the proliferation of CD122^+^ IELPs and on genes regulated during cell cycle, it is unlikely that this fully explains all differences between IELPs from TBGR *C-myc*^*fl/fl*^ and TBGR *C-myc*^*Δ/ΔCd4*^ mice. Recently, it has become clear that C-Myc acts as general amplifier that globally enhances transcription. For example, stimulation of resting B cells will result in the expression of C-Myc and its binding at nearly all promoters in open chromatin.^39^ Thus C-Myc exploits a pre-existent chromatin landscape for its actions.^40^ Additional regulators and C-Myc-binding partners will then define the ultimate transcriptional outcome. These far-reaching actions of C-Myc were also reflected in our scRNA-seq data from TBGR *C-myc*^*Δ/ΔCd4*^ IELPs, which clearly demonstrated that C-Myc not only regulates cell cycle but also has a profound impact on energy metabolism and overall protein synthesis. C-Myc deregulation will therefore have complex effects in various cell types and cannot be anticipated based on purely linear models. As a result of these wide-reaching effects, we saw a separate clustering of CD122^+^T-bet^−^ NK1.1^−^ IELPs from TBGR *C-myc*^*Δ/ΔCd4*^ mice when compared with TBGR *C-myc*^*fl/fl*^ mice. Moreover, we provide evidence that C-Myc plays an important role in the context of agonist selection, as NK1.1^−^ IELPs from TBGR *C-myc*^*Δ/ΔCd4*^ mice fail to further differentiate.

Despite decades of research and their abundance in the intestinal tract, natural IELs are still enigmatic. Here we provide a detailed molecular road map of the thymic development of NK1.1^−^ IELPs, which will enable us to better understand the step-wise differentiation of these cells. This might pave the way to new models that could assign unique and important functions to these cells.

## Materials and methods

### Mouse strains

*Rosa26R*^*Yfp/Yfp*^, *Id2*^*CreERT2*^,^41^ *Cd4*^*Cre*^^-^^*Tg*^;*C-myc*^*fl/fl*^ (provided by Anne Wilson, Lausanne, Switzerland),^19^ *Tbx21*^ZsGreenTg^ (TBGR mice),^23^ *Act-mOVA*,^42^ *OT-1*,^43^ *Id3*^*Gfp/+*^*,*^29^ and *Id2*^*Gfp/+*^ ^41^ mice on a C57BL/6 background were bred locally. TBGR mice were crossed to C57BL/6 (purchased from Janvier Laboratories) mice and all other mouse strains were crossed to heterozygous or homozygous littermates. For all experiments, both male and female littermate mice were used as a control and mice were between 4 and 16 weeks of age if not otherwise indicated and group caged. All animal experiments were approved and are in accordance with the local animal care committees and Regierungspräsidium Freiburg.

### Isolation of IELs

Isolation of IELs was carried out as previously described.^14^ In brief, the proximal intestine was removed by mechanical dissociation, eliminated from fat tissue and Peyer’s patches. Tissue was opened longitudinally, cut into small pieces of 3–4 cm, and washed with ice-cold phosphate-buffered saline (PBS). Epithelial cells were removed by incubation twice for 20 min at 37 °C in cell-separation Hanks’ Balanced Salt Solution (Sigma-Aldrich, St. Louis, MO) medium containing 5 mM EDTA, 1 mM dithiothreitol (American, Bioanalytical, Natick, MA), and 10 mM Hepes, followed by vortexing for 30 s and filtered through a 70-µm cell strainer. Leukocytes were enriched by performing 40%/80% Percoll (Sigma-Aldrich) gradient centrifugation.

### Isolation of IELPs

IELPs were isolated by dissecting the thymus and single-cell suspension of thymocytes was isolated by mashing the tissue through a 70-μm cell strainer (BD Biosciences, San Jose, CA) with 15 ml of ice-cold PBS. Lymphocytes of thymus were enriched by removing erythrocytes through incubation in red cell lysis buffer.

In case of further IELP enrichment, CD4^+^ and CD8^+^ thymocytes were depleted by incubation with purified anti-CD4 (GK1.5) and anti-CD8β (YTS156.7.7) antibody (both from BioLegend, San Diego, CA) and anti-mouse Dynabeads (Life Technologies, Carlsbad, CA) according to the manufacturer’s protocol.

### Isolation of splenocytes and hepatic lymphocytes

Splenocytes and hepatocytes were isolated by mashing the tissue trough a 70-μm cell strainer (BD Biosciences). Hepatic lymphocytes were further enriched by performing 40%/60% Percoll gradient centrifugation. Lymphocytes of spleen and liver were further enriched by removing erythrocytes.

### Flow cytometric analysis and cell sorting

After generation of single-cell suspensions, the cells were incubated on ice for 30 min with anti-CD16/CD32 antibody for FC receptor blocking and stained with fluorescent-label coupled antibodies (BioLegend) in PBS (Ca2^+^ and Mg2^+^-free) supplemented with 2 mM EDTA and 2% fetal calf serum. The following conjugated antibodies were used: CDε (145-2C11), CD4 (RM4-5), CD45.2(104), CD8α (53-6.7), CD8β (YTS156.7.7), H2-K^b^ (AF6-88.5), TCRγδ (GL3), TCRβ(H57-597), TCR Vα2 (B20.1), TCR Vβ5 (MR9-4), CD90.1 (Thy-1.1, OX-7) CD90.2 (Thy-1.2; 30H12), CD8β (YTS156.7.7), NK1.1 (PK136), CD122 (TM-β1), CD24 (M1/69), CD25 (PC61), CD5 (53-7.3), CD44 (IM7), CD62L (MEL-14), PD-1 (29.F1A12), Qa-2 (695H1-9-9), CD49a (HMα1), CD49b (DX5). T-bet (4B10) was stained by using the FoxP3 TF staining buffer set (Thermo Fisher Scientific) and stained on ice for 2 h with TF antibodies. In order to stain fluorescent-reporter mice in combination with TFs, cells were fixated with Cytofix/Perm (BD Biosciences) for 20 min after surface staining, washed twice in wash buffer, and incubated with anti-GFP (Life Technologies) for 1 h. After washing, cells were fixated overnight in FoxP3 TF staining buffer set (Thermo Fisher Scientific), washed twice, TF were stained in FoxP3 TF Perm/Wash buffer for 2 h, and analyzed on the next day.

For CD1d tetramer staining, single-cell suspension was first stained with CD1d tetramer allophycocyanin-conjugated or unconjugated antibodies (Proimmune, Oxford, UK) for 30 min at 4 °C in dark, washed by centrifugation at 1500 rpm for 5 min at 4 °C, and stained with surface antibodies as described before.

Cells were analyzed on a BD LSRFortessa (BD Bioscience) flow cytometer and analyzed with the FlowJo software (Treestar, Ashland, OR).

In case of cell sorting, single-cell suspension of the thymus was stained with fluorescent-label coupled antibodies in PBS (Ca^2+^ and Mg^2+^-free) supplemented with 2 mM EDTA and 1% bovine serum albumin (Sigma-Aldrich) and then sorted (purity > 98%) by using a BD FACSAria III cell sorter (BD Biosciences). After sorting, plates were centrifuged for 5 min at 2200 g at 4 °C, snapfrozen in liquid nitrogen and stored at −80 °C until processed.

### Single-cell RNA amplification and library preparation

αCD4- and αCD8-depleted IELPs were FACS (fluorescence-activated cell sorting) sorted into 384-well plates on a BD Influx cell sorter using single-cell mode and influx information was recorded. scRNA-seq was performed using the CEL-Seq2 method^44^ with several modifications. A fivefold volume reduction was achieved using a nanoliter-scale pipetting robot (Mosquito HTS, TTP Labtech). IELPs were sorted into 384-well plates containing 240 nl of primer mix and 1.2 μl of PCR encapsulation barrier, Vapor-Lock (QIAGEN, Hilden, DE). Sorted plates were centrifuged at 2200 × *g* for few minutes at 4 °C, snap-frozen in liquid nitrogen, and stored at −80 °C until processed. One hundred and sixty nanoliters of RT reaction mix and 2.2 μl of second strand reaction mix were used to convert RNA into cDNA. cDNA from 96 cells was pooled together before clean up and in vitro transcription, generating 4 libraries from one 384-well plate. In all, 0.8 μl of AMPure/RNAClean XP beads (Beckman Coulter) per 1 μl of sample were used during all the purification steps including the library clean up. Other steps were performed as described in the original protocol.^24^ IELPs were sequenced on an HiSeq sequencing system 2500 (Illumina, San Diego, CA) or Hiseq 3000 sequencing system (Illumina) for TBGR and TBGR *C-myc*^*Δ/ΔCd4*^ (pair-end multiplexing run, high output mode) at a depth of ~100,000–250,000 reads per cell.

### Quantification of transcript abundance

Paired end reads were aligned to the transcriptome using bwa (version 0.6.2-r126) with default parameters.^45^ The transcriptome contained all gene models based on the mouse ENCODE VM9 release downloaded from the UCSC genome browser comprising 57,207 isoforms with 57,114 isoforms mapping to fully annotated chromosomes (1–19, X, Y, M). All isoforms of the same gene were merged to a single gene locus. Furthermore, gene loci overlapped by >75% were merged to larger gene groups. This procedure resulted in 34,111 gene groups. The right mate of each read pair was mapped to the ensemble of all gene loci and to the set of 92 ERCC spike-ins in sense direction.^46^ Reads mapping to multiple loci were discarded. The left read contained the barcode information: The first six bases corresponded to the unique molecular identifier (UMI), followed by six bases representing the cell-specific barcode. The remainder of the left read contained a polyT stretch. The left read was not used for quantification. For each cell barcode, the number of UMIs per transcript was counted and aggregated across all transcripts derived from the same gene locus. Based on binomial statistics, the number of observed UMIs was converted into transcript counts^47^.

### Clustering

For 4-week-old TBGR mice 645 cells (Fig. 1) and for 2-week-old TBGR mice (including TBGR *C-myc*^*fl/fl*^ and TBGR *C-myc*^*Δ/ΔCd4*^ mice) 347 cells (Fig. 5) passed the quality threshold and 18,680 or 21,012 genes, respectively, were quantified across these cells, excluding mitochondrial genes and ERCC spike-ins. The datasets were analyzed using RaceID3^24^. Rescaling to 2,000 transcripts per cell was used for data normalization. Importantly, prior to normalization, cells expressing >2% of *Kcnq1ot1* transcripts, a previously identified marker of low quality cells were removed from the analysis^25^. Moreover, genes correlating to *Kcnq1ot1*, *Gm10715*, *Gm42418* and *Gm10800* with a Pearson’s correlation coefficient >0.65 were removed. RaceID3 was run with default parameters except for the following: mintotal = 2000, minexpr = 3, outminc = 3, FSelect = TRUE. Outlier identification was omitted. Only cells with at least mintotal transcripts (UMI count) were included in the analysis. To remove cell-cycle and batch associated variability the FGenes and CGenes parameter were initialized. CGenes = c("Pcna", "Mki67"); FGenes = c("Malat1", "Xist") with ccor = 0.4.

### Differential gene expression analysis

Differentially expressed genes between two subgroups of cells were identified similar to a previously published method.^48^ First, negative binomial distributions reflecting the gene expression variability within each subgroup were inferred based on the background model for the expected transcript count variability computed by RaceID3. Using these distributions, a *p* value was calculated for the observed difference in transcript counts between the two subgroups, multiple testing corrected by Benjamini–Hochberg method and referred to as "adjusted p-value".^24^

### Lineage inference and pseudo-temporal ordering

For derivation of IELP differentiation trajectories, the StemID2 algorithm was used.^24,25^ StemID2 was run with the following parameters: cthr = 5, pdishuf = 2000, pthr = 0.01, pethr = 0.01, nmode = TRUE. Pseudo-temporal order was derived based on StemID2 projection coordinates of highest projection of cell to medoid links onto inter-cluster links of the subsequent cluster along the selected trajectory (5>3>1>4>2). Self-organizing maps (SOMs) were used to infer models of pseudo-temporal expression profiles using the following parameters: ksom = 20, nbsom = 1000, alpha = 0.5, corthr = 0.85, minsom = 5.

### BrdU staining

Mice were injected with 1 mg/200 µl BrdU intraperitoneally and BrdU was incorporated for 3 h. Mice were scarified and the organs of interest isolated. BrdU labeling was analyzed according to the manufacturer’s instructions (BD Pharmingen). Cell were analyzed on a BD LSRFortessa (BD Bioscience) flow cytometer and analyzed with the FlowJo software (Treestar).

### Generation of bone marrow chimeras

*Act-mOVA* mice (CD90.2) were lethally irradiated with 12 Gy and reconstituted with T cell-depleted bone marrow from either *C-myc*^*fl/fl*^ OT1 or *C-myc*^*fl/fl*^ *Cd4*^*Cre-Tg*^ OT1 (CD90.1) mice. All irradiated mice received antibiotics in the drinking water (1 g/l neomycin sulfate, Thermo Fisher Scientific) during the first 3 weeks after irradiation.

### Quantitative real-time PCR

Lymphocyte populations of interest were FACS sorted and collected. Total RNA was isolated by using TRIZOL (Life Technologies) according to the manufacturer’s instructions. RNA concentrations were determined using Nanodrop 1000 (Thermo Fisher Scientific). RNA was reverse transcribed using the High Capacity cDNA Reverse Transcription Kit (Thermo Fischer) according to the manufacturer’s protocol (Applied Biosystems). qPCR was performed using SyberGreen Gene Expression Assay (Life Technologies) with the following primers: *C-myc* forward 5’-GCCCCCAAGGTAGTGATCCT-3’ and reverse 5’-GTGCTCGTCTGCTTGAATGG-3’ and *Id3* forward 5’-GAAATCCTGCAGCGTGTCAT-3’ and reverse 5’-GTCAGTGGCAAAAGCTCCTC-3’. Gene expression was normalized as *n*-fold difference to the gene *Hprt1* forward 5’-TGATCAGTCAACGGGGGACA-3’ and reverse 5’-TTCGAGAGGTCCTTTTCACCA-3’. qPCR reaction was performed on a ABI Prism 7900 sequence detector (Applied Biosystems, Foster City, CA).

### Statistical analysis

*p* Value of datasets was determined by unpaired two-tailed Student’s *t* test with 95% confidence interval. All statistical tests were performed with the Graph Pad Prism V4 software (Graph Pad Software, La Jolla, CA). (*p < 0.05; **p < 0.01, and ***p < 0.001; n.s., not significant).

## Supplementary information

Supplementary Infoformation

## Data Availability

Single-cell RNA sequencing (scRNA-seq) datasets are available at NCBI GEO (GSE122740) or using the link https://www.ncbi.nlm.nih.gov/geo/query/acc.cgi?acc=GSE122740.
