# Supplementary material for: Single-cell RNA-sequencing identifies the developmental trajectory of C-Myc-dependent NK1.1− T-bet+ intraepithelial lymphocyte precursors
Source: Mucosal Immunol. 2019 Nov 11;13(2):257–70. doi: 10.1038/s41385-019-0220-y (PMC7039806; doi:10.1038/s41385-019-0220-y)
Supplement: Supplementary file 1 — Supplementary Infoformation [file 41385_2019_220_MOESM1_ESM.pdf]

# Single-cell RNA-sequencing identifies the developmental trajectory of C-Myc-dependent NK1.1$^{-}$ T-bet$^{+}$ intraepithelial lymphocyte precursors

## Supplementary information

**Authors:** Jonas F. Hummel[1], Patrice Zeis[2,3,4], Karolina Ebert[1], Jonas Fixemer[1], Philip Konrad[1], Christian Schachtrup[6], Sebastian J. Arnold[7], Dominic Grün[2,8] and Yakup Tanriver[1,5]

**Authors' affiliations:**

[1]Institute of Medical Microbiology and Hygiene, University Medical Center Freiburg, 79106 Freiburg, Germany

[2]Max Planck Institute of Immunobiology and Epigenetics, 79108 Freiburg, Germany

[3]Faculty of Biology, University of Freiburg, Schaenzlestrasse 1, 79104 Freiburg, Germany

[4]International Max Planck Research School for Molecular and Cellular Biology (IMPRS-MCB), Freiburg, Germany

[5]Department of Internal Medicine IV, University Medical Center Freiburg, 79106 Freiburg, Germany

[6]Institute of Anatomy and Cell Biology, Faculty of Medicine, University of Freiburg, 79104 Freiburg, Germany

[7]Institute of Experimental and Clinical Pharmacology and Toxicology, Faculty of Medicine, University of Freiburg, 79104 Freiburg, Germany

[8]CIBSS - Centre for Integrative Biological Signaling Studies, University of Freiburg, Freiburg, Germany

**Corresponding author:**

Yakup Tanriver, M.D.

University Medical Center Freiburg,

Hugstetter Strasse 55

D-79106 Freiburg, Germany

Tel: +49 (761) 203 6522        Fax: +49 (761) 203 6651

Email: yakup.tanriver@uniklinik-freiburg.de

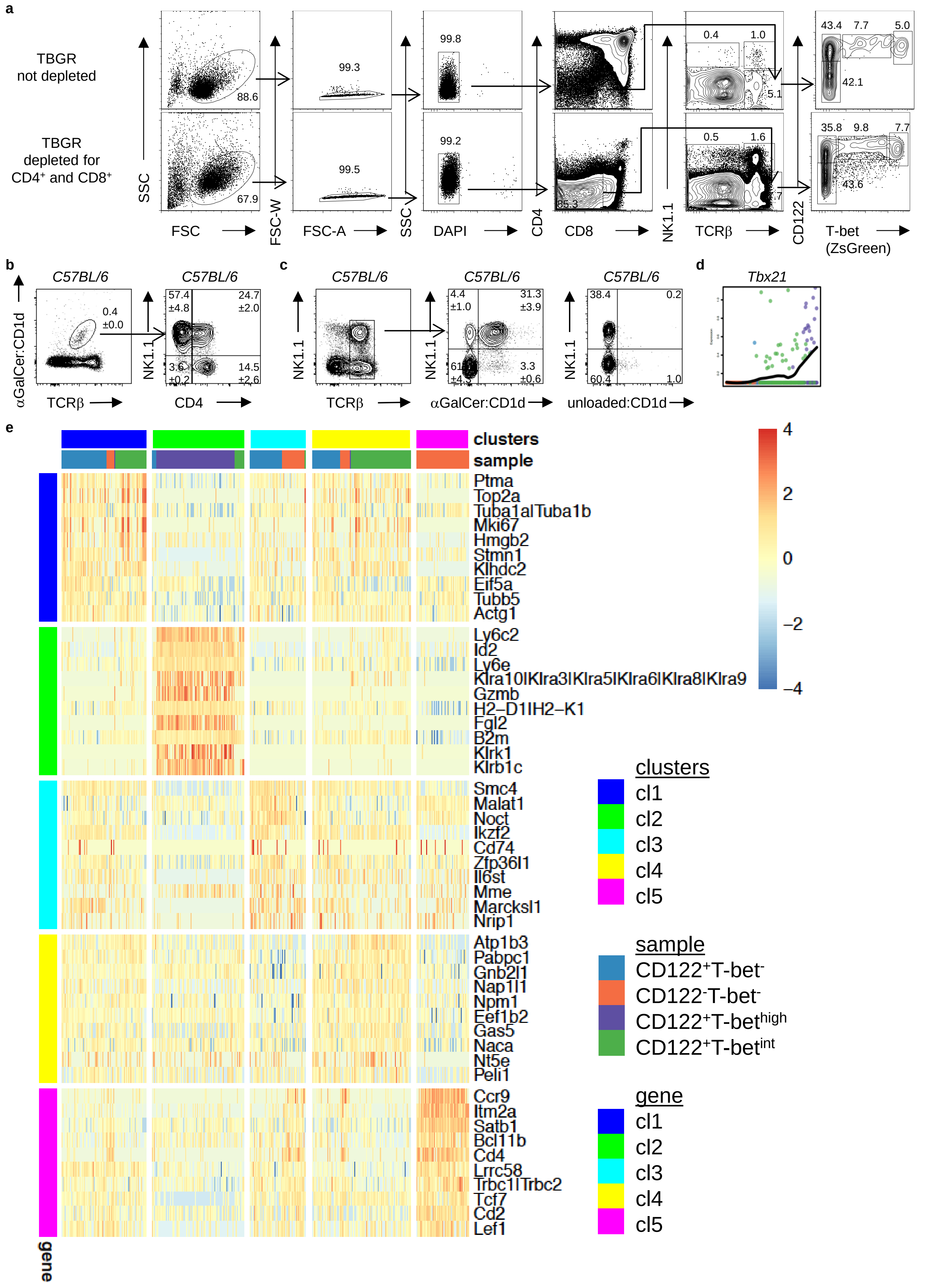


Supplement Figure 1

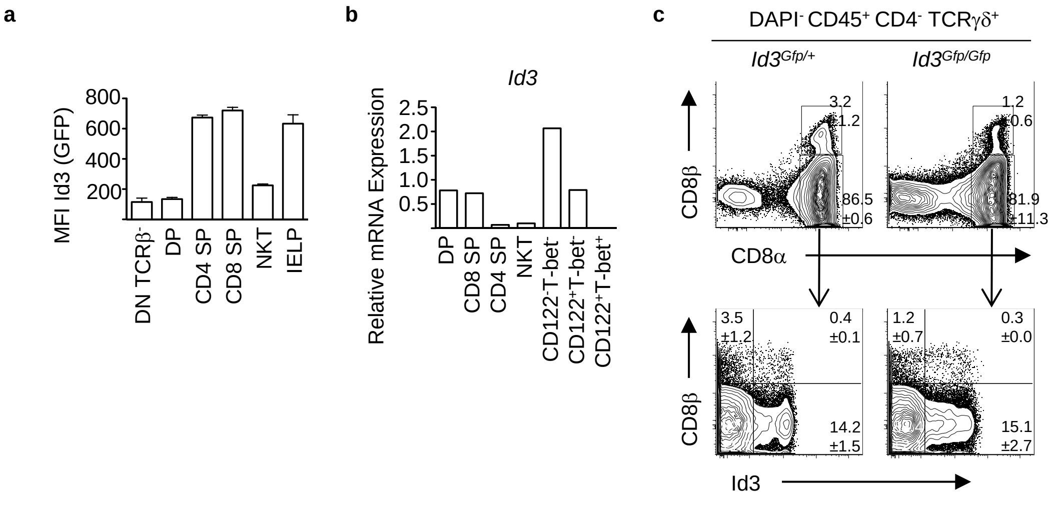


**Supplement Figure 2**

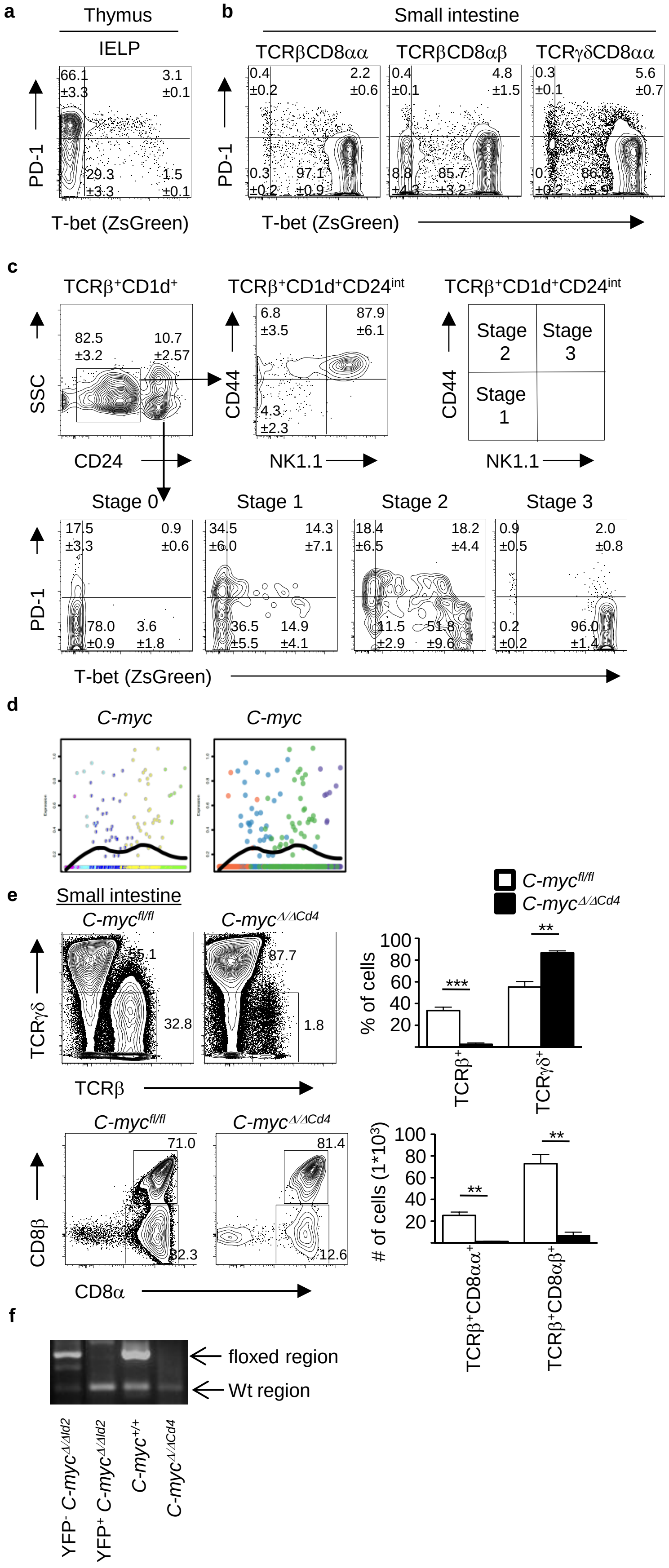


Supplement Figure 3

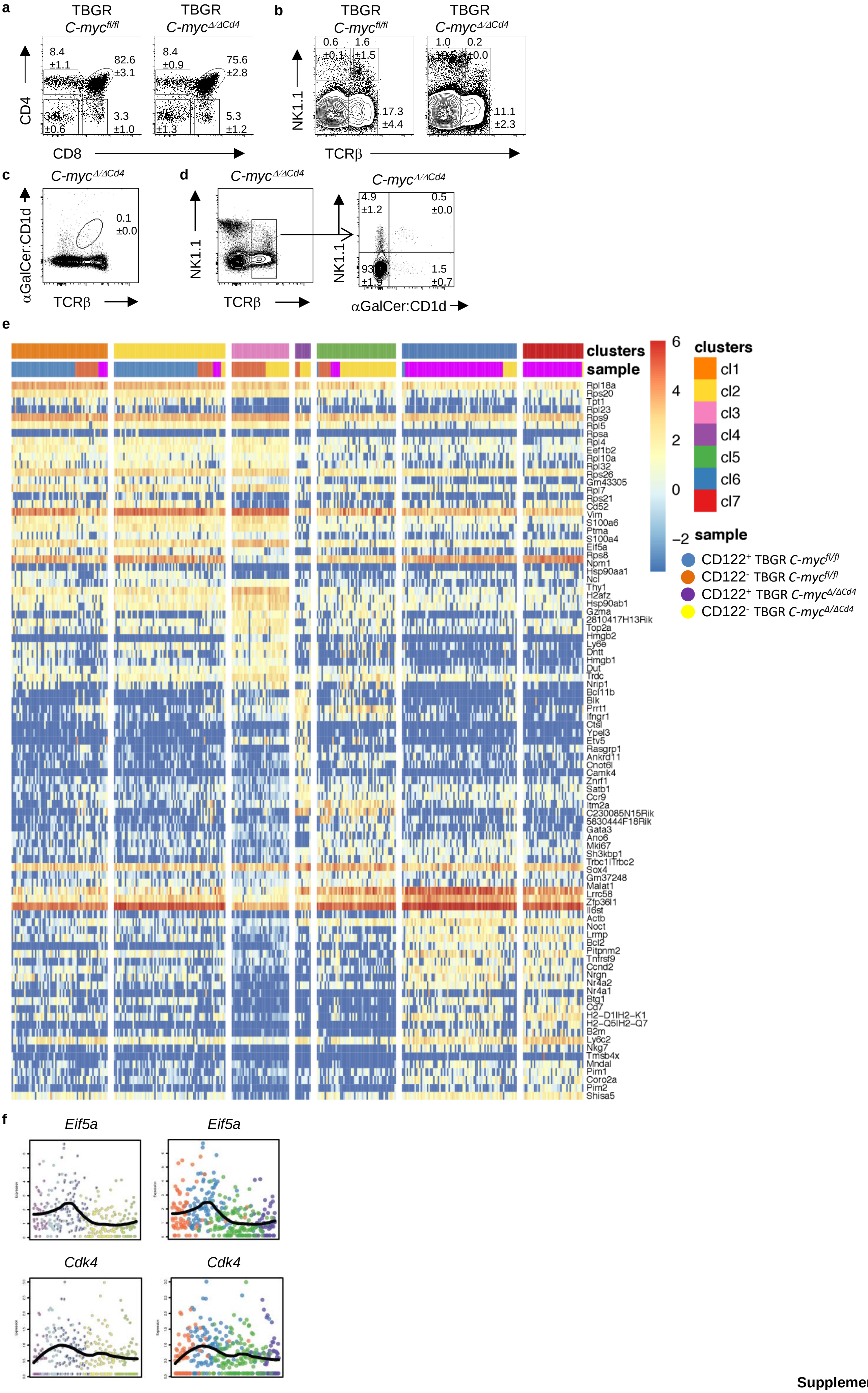


Supplement Figure 4

**Supplementary Figure 1: Single cell transcriptomics reveal thymic differentiation pathway of IELPs**

(a) Exemplary gating strategy of thymic flow cytometry analysis from TBGR mice (upper row). For cell sorting NK1.1$^-$ IELPs were enriched by depleting CD8 and CD4 by using Dynabeads magnetic isolation strategy (lower row). (b) Flow cytometric analysis of $\alpha$GalCer:CD1d expressing thymocytes in 4 week old *C57BL/6* mice (left panel). Numbers denote percentage of cells in gate (Mean ± SEM) for at least three mice. Further analysis of $\alpha$GalCer:CD1d expressing thymocytes for CD4 vs. NK1.1 expression (right panel). (c) DN TCR$\beta^+$ thymocytes (left panel) were analyzed for the expression of $\alpha$GalCer:CD1d vs. NK1.1 expression (middle panel). Unloaded:CD1d was used for setting gates (right panel). (d) *Tbx21* expression along the predicted IELP trajectory from Figure 1b with sample information. The black line indicates a local regression. (e) Heat map showing the centered and scaled log2-expression of the top ten differentially expressed genes (based on inferred adjusted p-value, adjusted p-value < 0.05, in increasing order of p-values) of the respective cluster (y-axis) across cell clusters 1 - 5 (x-axis). Log2 values above 4 or below -4 were set to 4 or -4, respectively. Differentially expressed genes were obtained by comparing gene expression of cells of the respective cluster to all other clusters and preserved if the respective gene is also differentially expressed in all pair-wise comparisons using clusters 1 - 5. *Rps*, *Rpl*, *Rik*, *Gm* and *RP* genes were removed if present. RaceID3 clustered cells are color-coded along the x-axis and the respective differentially expressed genes of each cluster along y-axis.

**Supplementary Figure 2: Id3 regulates the pool size of thymic IELPs**

(a) Bar diagram shows MFI of GFP (Id3) in different thymocyte subsets. (b) Bar diagram represents mRNA expression of *Id3* for different thymocyte subsets of three 6 - 12 week old TBGR mice performed by qPCR, experiments were performed twice, one representative result is shown. (c) Flow cytometry analysis of TCR$\gamma\delta^+$ IELs isolated from the epithelium of the small intestine from 6 - 12 week old $Id3^{Gfp/Gfp}$ mice with $Id3^{Gfp/+}$ littermates as controls as in Figure 2h. Cells were pre-gated for DAPI$^-$ CD45$^+$ CD4$^-$ TCR$\gamma\delta^+$.

**Supplementary Figure 3: Unconventional agonist-selected T cells progress through a PD-1 stage before up-regulating T-bet**

(a) Flow cytometry analysis of PD-1 vs. T-bet from thymic NK1.1$^-$ IELPs of 6 - 12 week old TBGR mice. Pre-gating is shown in Suppl. Figure 1 a. Numbers denote percentage of cells in the gate (Mean ± SEM) from at least 6 mice. (b) Flow cytometry analysis of TCRβ$^+$ and TCRγδ$^+$ lymphocytes isolated from the epithelium of the small intestine (IELs) of 6 - 12 week old TBGR mice pre-gated for DAPI$^-$ CD45$^+$ CD4$^-$; TCRαβ$^+$ CD8αα$^+$ (left panel), TCRαβ$^+$ CD8αβ$^+$ (middle panel) and TCRγδ$^+$ CD8αα$^+$ (right panel). IELs were analyzed for PD-1 vs. T-bet expression. Numbers denote percentage of cells in the gate (Mean ± SEM) from at least 3 mice. (c) Flow cytometry analysis of thymocytes from 6 - 12 week old TBGR mice pre-gated on CD1d$^+$ TCRβ$^+$ NKT cells. NKT cell development was divided into 4 stages based on SSC and the expression of CD24, CD44, NK1.1. Numbers denote percentage of cells in gate (Mean ± SEM) from 3 mice. (d) C-myc expression along the predicted IELP trajectory from Figure 1b with cluster information (left panel) or sample information (right panel). The black line indicates a local regression. (e) Upper row shows flow cytometry analysis of TCRβ$^+$ and TCRγδ$^+$ lymphocytes isolated from the epithelium of the small intestine (IELs) of 6 - 12 week old *C-myc*$^{fl/fl}$ (littermate control) and *C-myc*$^{\Delta/\Delta Cd4}$ mice pre-gated on DAPI$^-$ CD45$^+$ CD4$^-$. Numbers denote percentage of cells in gate (Mean ± SEM) from at least 6 mice per genotype. Lower row shows further analysis of TCRβ$^+$ IELs for CD8α vs. CD8β expression. Bar diagrams show percentage (Mean ± SEM) of TCRβ$^+$ and TCRγδ$^+$ lymphocytes (upper row) and absolute cell numbers of TCRβ$^+$ IELs (lower row, Mean ± SEM). (f) YFP$^-$ and YFP$^+$ TCRβ$^+$CD8αα$^+$ lymphocytes from small intestine of tamoxifen-treated *C-myc*$^{\Delta/\Delta Id2}$ mice and as controls TCRβ$^+$CD8αα$^+$ IELs from *C-myc*$^{+/+}$ and *C-myc*$^{\Delta/\Delta Cd4}$ mice were sorted by flow cytometry and tested for Cre-mediated excision of the floxed *C-myc* allele by PCR, experiments were performed thrice, one representative result is shown.

**Supplementary Figure 4: C-myc knockout NK1.1$^-$ IELPs show developmental arrest after agonist selection**

(a - b) Flow cytometric analysis of thymocytes from 6 - 16 week old TBGR *C-myc*$^{\Delta/\Delta Cd4}$ mice and TBGR *C-myc*$^{fl/fl}$ littermate mice as controls. Plots show thymic lymphocytes (CD45$^+$) (a) and DN thymocytes (b). Numbers denote percentage of cells in gate (Mean ± SEM) for at least 6 mice per genotype (c) Flow cytometric analysis of αGalCer:CD1d expression thymocytes in 2 - 3 week old *C-myc*$^{\Delta/\Delta Cd4}$ mice. Numbers denote percentage of cells in the gate (Mean ± SEM) for at least three mice. (d) DN TCRβ$^+$ thymocytes (left panel) were analyzed for the expression of αGalCer:CD1d vs. NK1.1 expression (right panel). Unloaded:CD1d was used for setting gates (not shown). (e) Heat map showing the log2-expression of the top 15 differentially expressed genes (based on inferred adjusted p-value, adjusted p-value $< 0.05$, in increasing order of p-values) of the respective cluster (y-axis) across cells of cluster 1 - 7 of RaceID3 inferred clustering (x-axis). Log2 values above 6 or below -4 are set to 6 or -4, respectively. Differentially expressed genes are obtained by comparing gene expression of cells of the respective cluster to all other clusters. RaceID3 clusters and the respective sample information are color-coded along the x-axis. (f) *Eif5a* and *Cdk4* expression along the predicted IELP trajectory from Figure 1b with cluster information (left panel) or sample information (right panel). The black line indicates a local regression.
